# SLC7A5 promotes colorectal cancer liver metastasis by reprogramming tryptophan metabolism through the Kyn/XANA‒AhR axis and reshaping the immune microenvironment

**DOI:** 10.1002/ctm2.70766

**Published:** 2026-08-06

**Authors:** Yaohao Luo, Lei Li, Shanbao Li, Xinshuai Wang, Zeping He, Fangbin Song, Jun Qin, Jinyan Zhang, Junming Xu

**Affiliations:** ^1^ Department of General Surgery Shanghai General Hospital Shanghai Jiao Tong University School of Medicine Shanghai China

**Keywords:** AhR, Colorectal cancer, CRLM, EMT, Immune evasion, SLC7A5

## Abstract

**Background:**

Colorectal cancer (CRC) is a leading cause of cancer‐related death and is associated with high recurrence rates. Solute carrier family 7 member 5 (SLC7A5), a core transporter that facilitates the transmembrane movement of tryptophan, plays a role in various cancers. However, whether and how SLC7A5 promotes colorectal liver metastasis (CRLM) through tryptophan metabolism reprogramming and immune remodelling remain unexplored.

**Methods:**

We integrated public datasets and clinical specimens to analyse SLC7A5 expression and prognosis, and validated its role in proliferation and metastasis using in vitro assays and in vivo models. Targeted metabolomics and isotope tracing identified kynurenine (Kyn) and xanthurenic acid (XANA) as downstream metabolites of SLC7A5. Single‐cell RNA sequencing (scRNA‐seq) and conditioned medium experiments were used to assess the impact of SLC7A5 on the tumour immune microenvironment (TIME).

**Results:**

SLC7A5 expression increases sequentially in normal tissue, primary tumours and liver metastases, and higher SLC7A5 levels are associated with worse prognosis. SLC7A5 facilitates CRC cell growth, metastasis and epithelial‒mesenchymal transition (EMT) by promoting the production of Kyn and XANA and subsequent activation of the aryl hydrocarbon receptor (AhR). scRNA‐seq analysis and conditioned medium experiments demonstrated that SLC7A5 knockdown reprograms the TIME by driving macrophages towards an antigen‐presenting phenotype, alleviating CD8^+^ T‐cell exhaustion, polarising CD4^+^ T cells towards Th1/Th17 subsets and triggering antigen‐driven immunoglobulin G (IgG)‐secreting B‐cell clonal expansion, effects that were reversed by exogenous Kyn/XANA supplementation. Moreover, combination therapy with the SLC7A5 inhibitor JPH203 and anti‐programmed cell death protein 1 (PD‐1) antibody produced synergistic tumour growth inhibition and heightened antitumour immune responses.

**Conclusions:**

Collectively, our findings reveal that SLC7A5 drives CRLM through tryptophan/Kyn/XANA–AhR signalling and concomitant remodelling of the TIME, positioning SLC7A5 as a promising target for combination therapy with anti‐PD‐1 in CRLM.

## Key points

- SLC7A5‒Kyn/XANA signalling activates AhR to drive EMT and metastatsis.
- SLC7A5 knockdown enhances macrophage antigen presentation and reverses CD8^+^ T‐cell exhaustion.
- SLC7A5 knockdown induces antigen‐driven and IgG‐secreting B‐cell clonal expansion.
- Combination of SLC7A5 inhibition and anti‐PD‐1 therapy offers clinical targeting.

## Graphical abstract

1. SLC7A5‒Kyn/XANA signalling activates AhR to drive EMT and metastatsis.
2. SLC7A5 knockdown enhances macrophage antigen presentation and reverses CD8^+^ T‐cell exhaustion.
3. SLC7A5 knockdown induces antigen‐driven and IgG‐secreting B‐cell clonal expansion.
4. Combination of SLC7A5 inhibition and anti‐PD‐1 therapy offers clinical targeting.

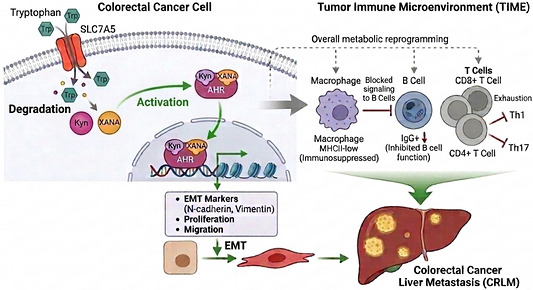

## 1 INTRODUCTION

Colorectal cancer (CRC) is the third most common cancer in the world.^1^ Its high recurrence and metastasis rates are the main reasons for the low survival rate of patients with CRC.^2^, ^3^, ^4^ However, patients with colorectal liver metastasis (CRLM) have limited survival benefits because of the immunosuppressive tumour immune microenvironment (TIME) and amino acid metabolic reprogramming. Therefore, clarifying the relationships between CRLM and these two factors is important.

The development of a tumour is highly dependent on metabolic reprogramming, and the uptake and utilisation of amino acids is a key rate‐limiting step. Solute carrier family 7 member 5 (SLC7A5), a core transporter that facilitates the transmembrane movement of crucial amino acids, such as leucine and tryptophan, has recently attracted considerable attention because of its dual regulatory effects on tumour metabolism and antitumour immunity. SLC7A5 promotes tumour cell proliferation by activating the mechanistic target of rapamycin (mTOR) signalling pathway and affects the effector function of CD8^+^ T cells through the modulation of tryptophan metabolism, establishing a critical molecular connection between tumour metabolism and the immune response. Abnormal overexpression of SLC7A5 has been observed in various malignant tumours, including lung, breast, prostate and glioblastoma cancers, and its expression levels are closely linked to tumour progression and unfavourable patient outcomes. Given the established correlation between SLC7A5 upregulation and poor prognosis or chemoresistance in CRC, coupled with the recognised role of tryptophan metabolism in shaping tumour immune responses, elucidating how SLC7A5 orchestrates immune resistance through metabolic reprogramming is critical for understanding its function in promoting CRLM and facilitating immune evasion.

This study aimed to investigate the oncogenic functions of SLC7A5 in vitro and in vivo, and to elucidate how SLC7A5 exerts immunomodulatory effects on the TIME through tryptophan metabolic reprogramming. We found that SLC7A5 was overexpressed in CRC and associated with poor prognosis. Functional experiments demonstrated that SLC7A5 promoted tumour cell proliferation, migration and epithelial‒mesenchymal transition (EMT) by promoting the production of kynurenine (Kyn) and xanthurenic acid (XANA) and subsequent activation of the aryl hydrocarbon receptor (AhR). Furthermore, SLC7A5 knockdown remodelled the TIME by driving macrophages towards an MHCII^+^ antigen‐presenting phenotype, reversing CD8^+^ T‐cell exhaustion, and inducing immunoglobulin G (IgG)‐secreting B‐cell clonal expansion. Combination therapy with the SLC7A5 inhibitor JPH203 and anti‑programmed cell death protein 1 (PD‑1) antibody synergistically suppressed tumour growth and enhanced antitumour immunity. Thus, our study identifies SLC7A5 as a dual‐function metabolic checkpoint driving CRLM progression and a promising target for combination therapy with anti‐PD‐1.

## 2 MATERIALS AND METHODS

### 2.1 Public data resources and descriptions

The RNA sequencing data and clinical characteristics were obtained from The Cancer Genome Atlas (TCGA), Gene Expression Omnibus (GEO) and TNMplot database, which was further used for Gene Ontology (GO) analysis, gene set enrichment analysis (GSEA) and Kyoto Encyclopedia of Genes and Genomes (KEGG) analysis.

### 2.2 Patient specimens

Experiments involving patient specimens were approved by the Institutional Ethics Committee of Shanghai General Hospital (2024SQ521) with written informed consent. CRC specimens and paired normal mucosa were obtained from patients undergoing curative resection at Shanghai General Hospital between January 2018 and December 2020, and a tissue microarray (TMA) was constructed using 80 paired normal and tumour tissues. Inclusion criteria were histologically confirmed CRC, no preoperative chemotherapy or radiotherapy, and available complete clinicopathological data. Exclusion criteria included familial adenomatous polyposis, Lynch syndrome and inflammatory bowel disease‐associated CRC.

### 2.3 Animals use and care

All animal procedures were approved by the Institutional Animal Care and Use Committee of Shanghai General Hospital (2025AW008). Male BALB/c mice (6–8 weeks old) were maintained under specific pathogen‐free conditions with regulated environmental parameters and had ad libitum access to food and water.

### 2.4 Haematoxylin and eosin, immunohistochemistry, immunofluorescence and multiplex immunohistochemistry

Paraffin‐embedded sections were dewaxed, rehydrated and stained with haematoxylin and eosin (H&E). For immunohistochemistry (IHC), sections underwent antigen retrieval, blocking of endogenous peroxidase and bovine serum albumin (BSA), followed by overnight incubation at 4°C with primary antibody and horseradish peroxidase (HRP)‐conjugated secondary antibody at 37°C. Staining was visualised with 3,3'‐diaminobenzidine (DAB) and counterstained with haematoxylin. For cell‐based immunofluorescence (IF), cells were fixed, permeabilised, blocked and incubated with anti‐AhR antibody overnight at 4°C, then with fluorescein isothiocyanate (FITC)‐conjugated secondary antibody (Solarbio) and 4',6‐diamidino‐2‐phenylindole (DAPI). Images were captured under standardised settings. Antibody details are listed in Table S2. For multiplex immunohistochemistry (mIHC), a multiplex IHC kit (Cat# abs50015; Absin) was used. For example, in the IL‐17/CD4/MHCII/F4/80 panel, primary antibodies were incubated sequentially (anti‐IL‐17, anti‐CD4, anti‐MHCII and anti‐F4/80), each followed by HRP‐conjugated secondary antibody and one tyramide signal amplification (TSA) cycle with Opal fluorophores, for a total of four TSA cycles. After the final cycle, sections were counterstained with DAPI and mounted with anti‐fade medium.

IHC‐stained slides were digitally scanned and independently scored by two pathologists blinded to group allocation using the *H*‐score system (0‒300). Inter‐observer agreement was confirmed (Pearson *r* > .9 for all markers) and mean scores were used for analysis. For cell‐based IF, mean fluorescence intensity was measured across the entire coverslip. For mIHC, whole‐slide images were quantified using QuPath v0.4.3 or inForm under blinded conditions, with results expressed as percentage of positive cells or fluorescence intensity, as specified in individual figure legends. Each data point represents one biological specimen.

For mIHC‐based spatial proximity analysis, whole‐slide images were quantified using QuPath v0.4.3. MHCII^+^ macrophages were defined as reference cells, and the density of IL‐17^+^ CD4^+^ T cells located within a 30‐µm radius was calculated and normalised to cells per mm^2^ of tissue area. Quantification was performed independently by two researchers blinded to group allocation, and statistical comparisons between groups were conducted using the Mann‒Whitney *U*‐test. Detailed results are provided in the corresponding figure legends.

### 2.5 Cell culture and establishment of stable cell lines

CRC cell lines (RKO, HCT8, HCT116, Lovo, SW620, CT26 and MC38) were purchased from American Type Culture Collection. All cells were maintained in Dulbecco's Modified Eagle Medium (DMEM) supplemented with 10% foetal bovine serum (FBS, Gibco) and 1% penicillin‒streptomycin at 37°C in a 5% CO_2_ atmosphere. Cell line authenticity was verified by short tandem repeat (STR) profiling, and routine mycoplasma testing confirmed the absence of contamination. Stable SLC7A5‐knockdown cells and AhR‐knockout cells were generated using lentiviral shRNA and sgRNA vectors, respectively (Genomeditech). SLC7A5‐overexpressing RKO cells were established by cloning the human SLC7A5 coding sequence (RefSeq: NM_003486.7) into the pLVX‐puro vector. Transduced cells were selected with puromycin. Overexpression of SLC7A5 was confirmed by Western blot; knockdown and knockout efficiency were validated by quantitative reverse transcription polymerase chain reaction (qRT‐PCR) and Western blot. shRNA and sgRNA sequences are listed in Table S3.

### 2.6 Subcutaneous tumour, CRLM and lung metastasis models in mouse

Ten C57 mice were subcutaneously injected with 5 × 10^6^ lentivirus‐transduced MC38 cells to establish the subcutaneous tumour model. Tumour volume was measured with calipers every three days over a 14‐day period, after which tumours were excised for weighing and imaging. For the CRLM model, anaesthetised nude mice or BALB/c mice received intrasplenic injection of 2 × 10^6^ lentivirus‐transduced RKO or CT26 cells, respectively. In the combination therapy experiment, starting on day 4 post‐inoculation, mice were divided into four groups and received intraperitoneal injections of vehicle, 10 µg/mouse anti‐PD‐1 antibody (clone RMP1‐14, Bio X Cell) alone, 10 mg/kg JPH203 (Sparkjade) alone, or 10 µg/mouse anti‐PD‐1 antibody plus 10 mg/kg JPH203 every other day for 28 days. For the experimental lung metastasis model, 2 × 10^6^ RKO cells stably expressing shSLC7A5 or shNC were delivered by tail vein injection. At 4 weeks post‐injection, livers and lungs were collected, weighed and imaged.

### 2.7 Bone marrow‐derived macrophage and T‐cell isolation, treatment and flow cytometry analysis

Bone marrow mononuclear cells (BMMNCs) were harvested from the femurs and tibias of 6–8‐week‐old BALB/c mice by Ficoll gradient centrifugation. After erythrocyte lysis, BMMNCs were cultured in DMEM supplemented with 10% FBS and 20 ng/mL recombinant murine macrophage colony stimulating factor (PeproTech) for 7 days to differentiate into bone marrow‐derived macrophages (BMDMs), with fresh medium replaced every 2–3 days. Differentiated macrophages were treated with conditioned medium from shNC or shSLC7A5 CT26 cells for 48 h. For rescue experiments, 50 µM Kyn (MedChemExpress) and 50 µM XANA (MedChemExpress) were added to the shSLC7A5 conditioned medium. After treatment, macrophages were collected and stained with anti‐mouse F4/80 (BioLegend), anti‐mouse CD86 (BioLegend) and anti‐mouse CD163 (BioLegend). Flow cytometry analysis was performed to assess the proportions of CD86‐positive and CD163‐positive macrophages.

Single‐cell suspensions of splenocytes were prepared from 6–8‐week‐old BALB/c mice. CD8^+^ T cells were enriched by positive selection using a CD8a^+^ T‐cell isolation kit (Miltenyi Biotec). Purity was confirmed by flow cytometry (>90% CD8a^+^). CD8^+^ T cells were activated with plate‐bound anti‐mouse CD3 (5 µg/mL, BioLegend) and soluble anti‐mouse CD28 (5 µg/mL, BioLegend) in the presence of recombinant murine interleukin‐2 (IL‐2; 40 ng/mL, BioLegend) in RPMI 1640 medium supplemented with 10% FBS for 48 h. Activated CD8^+^ T cells were then treated with conditioned medium from shNC or shSLC7A5 CT26 cells for an additional 48 h. For rescue experiments, 50 µM Kyn and 50 µM XANA were added to the shSLC7A5 conditioned medium. After treatment, cells were collected and stained with anti‐mouse CD8 (BioLegend), anti‐mouse PD‐1 (BioLegend) and anti‐mouse LAG3 (BioLegend). Flow cytometry analysis was performed to evaluate PD‐1 and LAG3 expression on CD8^+^ T cells.

To analyse tumour‐infiltrating immune cells, freshly harvested tumours were digested into single‐cell suspensions using a Tumour Dissociation Kit (Miltenyi Biotec) according to the manufacturer's protocol. CD45^+^ immune cells were then enriched by magnetic bead sorting and stained with anti‐mouse F4/80 (BioLegend), anti‐mouse CD86 (BioLegend), anti‐mouse CD163 (BioLegend), anti‐mouse CD8 (BioLegend), anti‐mouse PD‐1 (BioLegend) and anti‐mouse LAG3 (BioLegend) for flow cytometry analysis. All experiments were performed in triplicates.

### 2.8 Ultra performance liquid chromatography–tandem mass spectrometry quantification of tryptophan metabolites

Cell pellets were resuspended in ultrapure water, mixed with pre‐cooled methanol (‒20°C) and vortexed for protein precipitation. Cell lysis was accomplished by subjecting the samples to repeated freeze–thaw cycles in liquid nitrogen. After centrifugation, the supernatant was collected, incubated at ‒20°C to precipitate residual proteins and centrifuged again. The final supernatant was analysed by Ultra performance liquid chromatography–tandem mass spectrometry (UPLC‐MS/MS) to quantify tryptophan pathway metabolites.

### 2.9 Stable isotope tracing

shNC and shSLC7A5 RKO cells were cultured in complete medium supplemented with ^1^ ^3^C_11_‐tryptophan (MedChemExpress) for 48 h. Metabolites were extracted using 80% methanol and analysed by liquid chromatography–tandem mass spectrometry (LC‐MS/MS) as described above. Natural isotope abundance correction was performed using the accucor R package (resolution = 60 000) to obtain corrected isotopologue peak intensities and mass isotopologue distributions. The relative abundance of ^1^ ^3^C_10_‐Kyn (M+10) and ^1^ ^3^C_10_‐XANA (M+10) was used to quantify metabolic flux through the Kyn pathway.

### 2.10 Single‐cell RNA sequencing

On day 30, liver samples were collected: liver metastasis without SLC7A5 knockdown (NC_T, *n* = 5), paired paracancerous tissue (NC_PT, *n* = 5), liver metastasis with SLC7A5 knockdown (SH_T, *n* = 5) and paired paracancerous tissue (SH_PT, *n* = 5). Each single‐cell RNA sequencing (scRNA‐seq) group included five independent biological replicates (individual mice), providing biological rather than technical replication. Then, CD45^+^ cells were isolated by flow cytometry for 10× Genomics scRNA‐seq. Cells with <200 or >5000 genes, >20% mitochondrial reads, or <500 unique molecular identifiers (UMIs) were excluded. Potential doublets were identified and filtered out with DoubletFinder v2.0.3 under the assumption of a 5% doublet rate. Normalisation was performed using SCTransform, and sample integration was performed using Harmony v1.0. Cells were clustered using the Louvain algorithm at a resolution of .8. Differentially expressed genes were identified via the Wilcoxon rank‐sum test. Cell‒cell communication was inferred with CellChat v1.6.1 applying the triMean method, and statistically significant ligand‒receptor pairs were determined at *p* < .05 following 100 permutation tests. B cell receptor (BCR) clonotypes were determined based on matching V and J gene segments together with complementary determining region 3 (CDR3) nucleotide sequences sharing ≥85% identity, and clonotype diversity was assessed using the Shannon and inverse Simpson indices.

### 2.11 Statistical analysis

Routine statistical tests and graphical representations were performed using GraphPad Prism 10.0, and advanced bioinformatics analyses were conducted using R (version 4.5.2). Data from in vitro experiments represent a minimum of three biological replicates and are displayed as mean ± SD. For animal experiments, the number of mice per group is indicated in each figure legend. Each dot represents one biological specimen. Pairwise comparisons were performed with a two‐tailed Student's *t*‐test, and differences among multiple groups were evaluated by one‐way ANOVA. The correlation between SLC7A5 expression and clinicopathological variables was assessed using the chi‐squared test. Statistical significance was defined as *p* < .05 (n.s.: not significant; ^*^ *p* < .05; ^**^ *p* < .01; ^***^ *p* < .001).

### 2.12 Additional experimental procedures

All remaining experimental procedures are detailed in Supporting Information S1: Materials and Methods.

## 3 RESULTS

### 3.1 SLC7A5 is upregulated in patients with CRLM and is associated with poor prognosis

**FIGURE 1:**
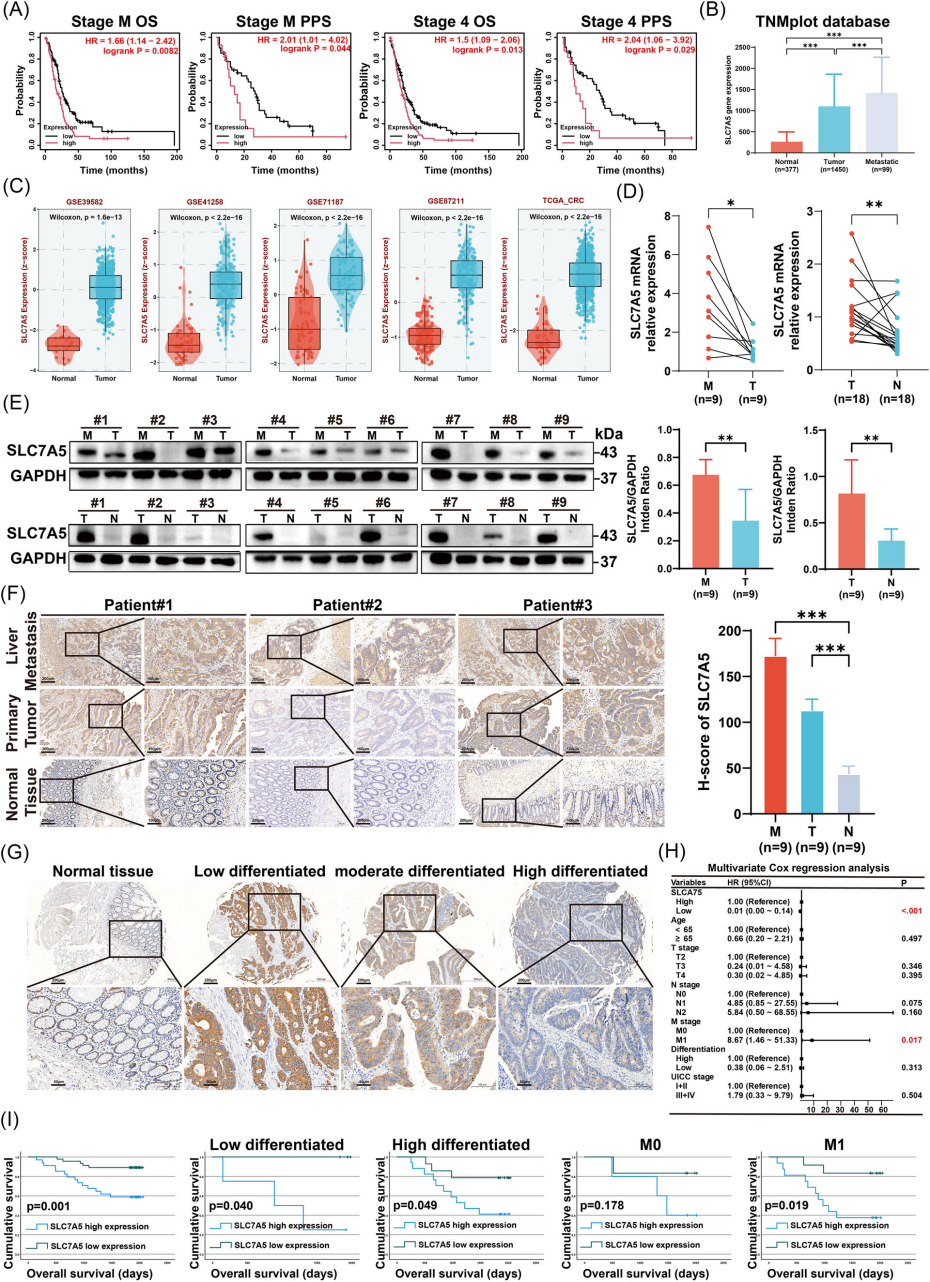
Solute carrier family 7 member 5 (SLC7A5) is upregulated in colorectal liver metastasis (CRLM) and associated with poor prognosis and immunosuppressive microenvironment. (A) Kaplan‒Meier survival curves stratified by SLC7A5 expression levels across different subgroups in The Cancer Genome Atlas (TCGA) and Gene Expression Omnibus (GEO) dataset. (B) SLC7A5 expression in metastatic and non‐metastatic colon adenocarcinoma samples from the Chip dataset in the TNMplot database. (C) Relative mRNA levels of SLC7A5 expression in liver metastasis and primary tumour, normal tissues from public CRLM dataset (TCGA_CRC, GSE87211, GSE39582, GSE41258, GSE71187). (D‒F) Relative mRNA levels, protein expression levels, and representative immunohistochemistry (IHC) images of SLC7A5 expression in liver metastasis and primary tumour, normal tissues from CRLM patients (*n*  = 9, scale bar = 200 or 100 µm). (G) IHC exhibits the expression of SLC7A5 in normal tissues and colorectal cancer (CRC) tissues with low, moderate and high differentiation (*n* = 80, scale bar = 200 or 50 µm). (H) Multivariate Cox regression analysis of OS in tissue microarray (TMA). (I) Kaplan‒Meier survival curves stratified by SLC7A5 expression levels in patients with low or high differentiated CRC, and in patients with or without tumour metastasis. Data are presented as mean ± SD, with individual data points shown. ^*^ *p* < .05, ^**^ *p* < .01, ^***^ *p* < .001.

Patients with advanced CRC and high SLC7A5 expression exhibited significantly reduced survival, as determined by analysis of the TCGA cohort (Figure 1A). To evaluate SLC7A5 expression across different lesion sites, we analysed a chip dataset from the TNMplot dataset and found that SLC7A5 expression increased sequentially in normal tissue, primary tumour and liver metastasis (Figure 1B), and analysis of GEO (GSE39582, GSE41258, GSE71187 and GSE87211) and the TCGA dataset revealed significantly higher SLC7A5 expression in primary tumours than in normal tissues (Figure 1C). Then, we collected clinical CRC samples to confirm the above results using qRT‐PCR, Western blotting and IHC (Figure 1D‒F). IHC staining of SLC7A5 protein in a TMA containing 80 cases of primary CRC paired with normal mucosa revealed that lower differentiation in CRC was associated with higher SLC7A5 expression, whereas SLC7A5 expression was substantially lower in adjacent normal mucosa than in CRC tissues (Figure 1G). Multivariate Cox regression analysis confirmed that high SLC7A5 expression was an independent predictor of poor overall survival (OS) (hazard ratio = .01, 95% confidence interval: .00‒.14, *p* < .001), after adjusting for age, T stage, N stage, M stage, tumour differentiation and Union for International Cancer Control (UICC) stage (Figure 1H). Additionally, OS analysis indicated that patients with CRC and high SLC7A5 expression had poor survival rates, irrespective of tumour differentiation level. Notably, among patients with stage M1 CRC, increased SLC7A5 expression was significantly correlated with shorter OS, whereas in patients with stage M0 CRC, elevated SLC7A5 expression did not significantly influence OS (*p* = .019 vs. *p* = .178) (Figure 1I). A similar trend towards shorter OS in patients with high SLC7A5 expression was also observed in those with stage III‒IV (compared with stage I‒II), T4 (compared with T2‒T3) and N1‒N2 (compared with N0) disease (Figure S1). Table 1 presents the correlation between SLC7A5 expression levels in the TMA and the clinicopathologic features of CRC patients. Elevated SLC7A5 expression was strongly associated with lymph node metastasis, distant metastasis, advanced UICC stage and tumour differentiation. By contrast, SLC7A5 expression was not significantly correlated with age, sex, tumour location, tumour size or depth of invasion (Table 1). Collectively, these findings suggest that elevated SLC7A5 expression may represent a novel independent prognostic indicator for poor OS.

**TABLE 1:** Association between solute carrier family 7 member 5 (SLC7A5) expression and clinicopathological features in colorectal cancer (*n* = 80).

Group	*N*	Low expression of SLC7A5 (*n* = 40)	High expression of SLC7A5 (*n* = 40)	*χ* ^2^	*p*‐Value
Age (years)				1.020	.313
<65	49	26 (53.1%)	23 (46.9%)		
≥65	31	20 (64.5%)	11 (35.5%)		
Gender				.019	.892
Male	44	25 (56.8%)	19 (43.2%)		
Female	36	21 (58.3%)	15 (41.7%)		
Tumour location				7.543	.056
Left hemicolon	13	11 (84.6%)	2 (15.4%)		
Right hemicolon	44	20 (45.5%)	24 (54.5%)		
Sigmoid colon	19	13 (68.4%)	6 (31.6%)		
Transverse colon	4	2 (50.0%)	2 (50.0%)		
Tumour size (cm)				3.040	.081
<6	42	28 (66.7%)	14 (33.3%)		
≥6	38	18 (47.4%)	20 (52.6%)		
T stage				3.639	.162
T2	8	7 (87.5%)	1 (12.5%)		
T3	30	15 (50.0%)	15 (50.0%)		
T4	42	24 (57.1%)	18 (42.9%)		
N stage				10.954	.004^a^
N0	50	35 (70.0%)	15 (30.0%)		
N1	19	9 (47.4%)	10 (52.6%)		
N2	11	2 (18.2%)	9 (81.8%)		
M stage				8.068	.005^a^
M0	69	44 (63.8%)	25 (36.2%)		
M1	11	2 (18.2%)	9 (81.8%)		
UICC stage				9.028	.029^a^
I	6	5 (83.3%)	1 (16.7%)		
II	38	26 (68.4%)	12 (31.6%)		
III	30	14 (46.7%)	16 (53.3%)		
IV	6	1 (16.7%)	5 (83.3%)		
Lymphatic vascular metastasis				6.215	.013^a^
No	48	33 (68.8%)	15 (31.2%)		
Yes	32	13 (40.6%)	19 (59.4%)		
Differentiation				9.378	.025^a^
Low	5	1 (20.0%)	4 (80.0%)		
Moderate‒low	8	3 (37.5%)	5 (62.5%)		
Moderate	60	35 (58.3%)	25 (41.7%)		
High‒moderate	7	7 (100.0%)	0 (0%)		

^a^Significant difference.

### 3.2 SLC7A5 promotes CRC cell proliferation and tumour growth

**FIGURE 2:**
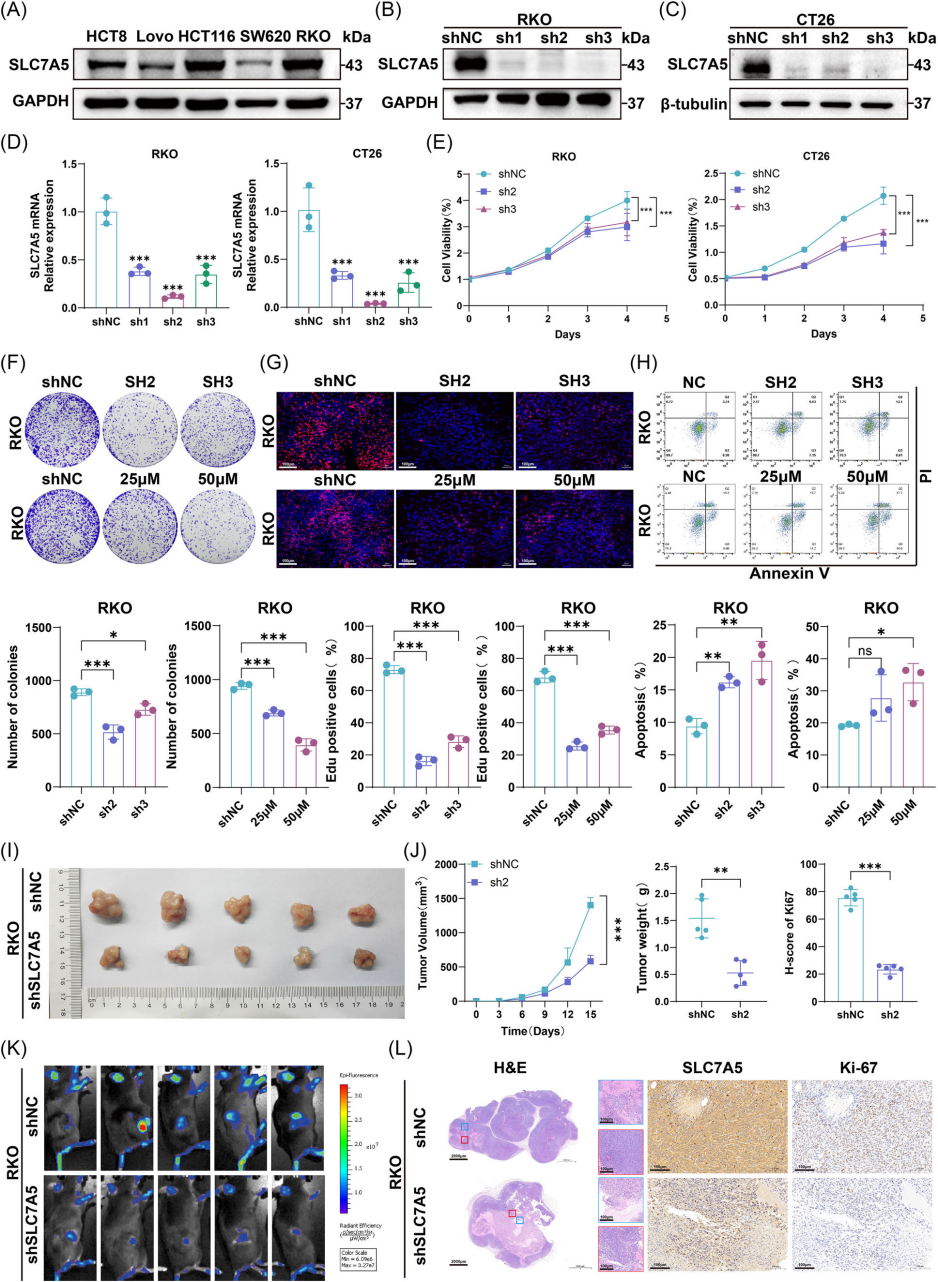
Solute carrier family 7 member 5 (SLC7A5) promotes colorectal cancer (CRC) cell proliferation and tumour growth. (A) Western blot analysis assessed SLC7A5 expression in HCT8, Lovo, HCT116, RKO and SW620 cells. (B‒D) Western blot and qRT‐PCR confirm the knockdown efficiency of SLC7A5 in RKO and CT26 cells. (E‒G) CCK‐8, colony formation and EdU assay was used to evaluate the proliferative capacity of the SLC7A5‐knockdown group and the BCH treatment group (25 and 50 µM) in CRC cells (*n* = 3, scale bar = 100 µm). (H) The apoptotic capacity of the SLC7A5‐knockdown group and the BCH treatment group (25 and 50 µM) in RKO and CT26 cells were detected by flow cytometry (*n* = 3). (I) Subcutaneous tumours were established by injecting shSLC7A5 MC38 cells in C57 mice (*n* = 5). (J) Volume, weight and Ki‐67 expression of subcutaneous tumours (*n* = 5). (K) Representative bioluminescence images of the subcutaneous tumours in shNC or shSLC7A5 groups (*n* = 5). (L) Haematoxylin and eosin (H&E) staining, SLC7A5 and Ki‐67 staining of subcutaneous tumours (*n* = 5, scale bar = 200 or 100 µm). Data are presented as mean ± SD, with individual data points shown. ^*^ *p* < .05, ^**^ *p* < .01, ^***^ *p* < .001.

To investigate the role of SLC7A5 in CRC progression, we analysed SLC7A5 expression in five human CRC cell lines and established stable SLC7A5‐knockdown (shSLC7A5) cell lines in RKO and CT26 cells via lentiviral transduction (Figure 2A‒D). Cell Counting Kit‐8 (CCK‐8), colony formation and 5‐ethynyl‐2'‐deoxyuridine (EdU) assays revealed that SLC7A5 knockdown significantly inhibited cell proliferation and colony formation, whereas flow cytometry revealed increased apoptosis. Furthermore, 2‐Amino‐2‐norbornanecarboxylic acid (BCH), a selective SLC7A5 inhibitor, suppressed the proliferation of RKO and CT26 cells (Figures 2E‒H and S2A‒C). In a mouse subcutaneous tumour model, knockdown of SLC7A5 resulted in decreased tumour volume, weight and bioluminescence intensity, accompanied by a significant reduction in Ki‐67 expression (Figure 2I‒L). These findings support the conclusion that SLC7A5 drives CRC cell growth in culture and in animal models.

### 3.3 SLC7A5 promotes CRC cell migration, invasion, EMT and CRLM formation

**FIGURE 3:**
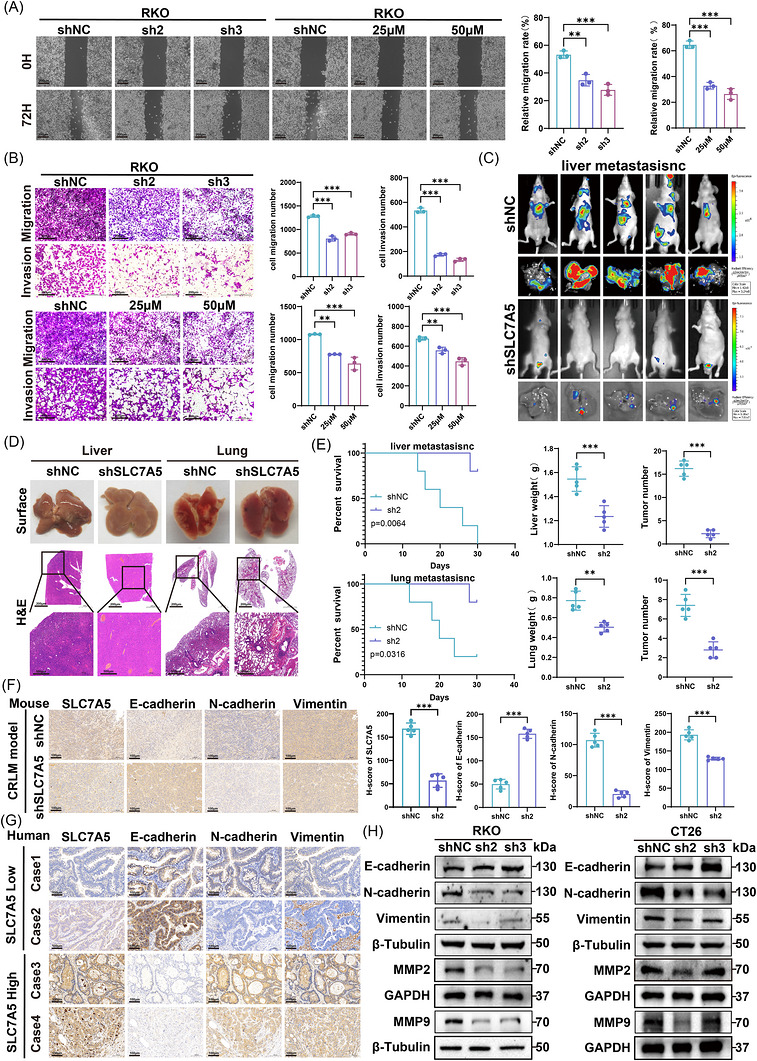
Solute carrier family 7 member 5 (SLC7A5) promotes colorectal cancer (CRC) cell migration, invasion, epithelial‒mesenchymal transition (EMT) and colorectal liver metastasis (CRLM) formation. (A and B) Wound healing and Transwell assays were used to evaluate the effects of SLC7A5 knockdown and BCH treatment (25 and 50 µM) on the migration of RKO and CT26 cells (*n* = 3, scale bar  =  200 µm). (C) Representative bioluminescence images of the livers in the CRLM model (*n* = 5). (D and E) Representative images of the constructed CRLM model and haematoxylin and eosin (H&E) staining of liver metastasis sections from RKO cells in different groups, including corresponding overall survival, liver weights and percentage of metastatic lesions (*n* = 5, scale bar = 200 or 500 µm). (F) Immunohistochemistry (IHC) analysis was used to evaluate the expression of E‐cadherin, N‐cadherin and Vimentin in CRLM model with different SLC7A5 levels (*n* = 3, scale bar = 100 µm). (G) IHC staining was performed to assess the expression of EMT‐related proteins in CRC patient samples (*n* = 5, scale bar = 100 µm). (H) Western blot analyses were performed to assess the expression of EMT‐related proteins in CRC cell lines and patient samples. Data are presented as mean ± SD, with individual data points shown. ^*^ *p* < .05, ^**^ *p* < .01, ^***^ *p* < .001.

To explore how SLC7A5 expression influences the invasive and metastatic behaviour of CRC, we performed a panel of functional studies. Both wound healing and Transwell assays consistently showed that SLC7A5 depletion or BCH treatment significantly impaired cell motility and invasiveness in RKO and CT26 lines (Figure 3A,B). In a nude mouse model of liver and lung metastasis, SLC7A5 knockdown decreased tumour bioluminescence, metastatic nodules, liver weight and EMT while prolonging survival (Figures 3C‒F and S3A,B). In patient samples and human CRC cell lines, Western blot analysis and IHC demonstrated that specimens or cells with decreased SLC7A5 expression presented increased levels of E‐cadherin and reduced levels of N‐cadherin and Vimentin. Additionally, the levels of MMP2 and MMP9, which are critical factors in extracellular matrix breakdown, as well as the levels of the EMT transcription factors Snail and Slug, were notably reduced in SLC7A5‐knockdown CRC cells (Figures 3G,H and S3C). These findings suggest that SLC7A5 facilitates the progression of EMT and CRLM.

### 3.4 Tryptophan metabolites Kyn and XANA activate AhR to promote CRLM

**FIGURE 4:**
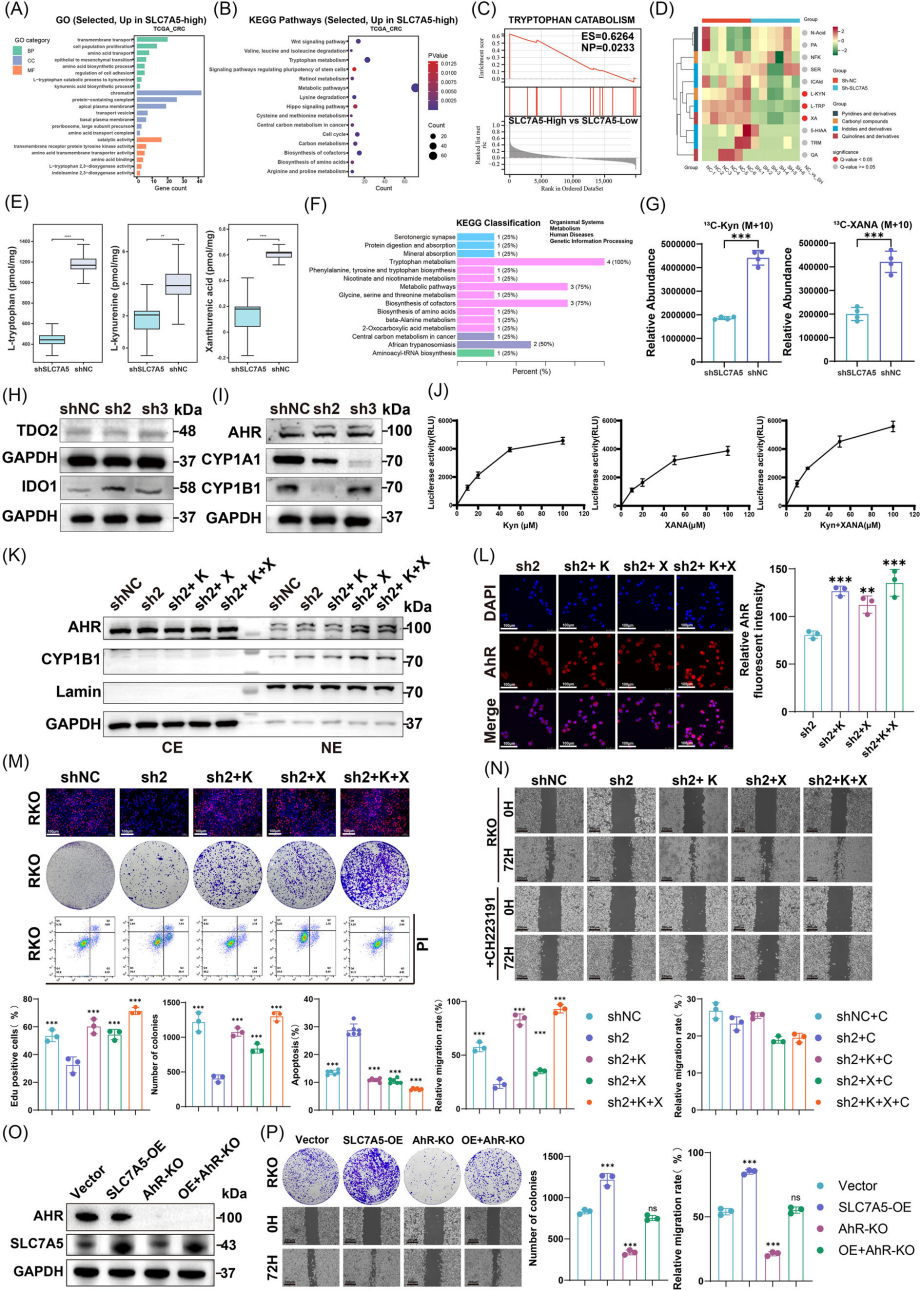
Tryptophan metabolites kynurenine (Kyn) and xanthurenic acid (XANA) activate aryl hydrocarbon receptor (AhR) to promote colorectal liver metastasis (CRLM). (A and B) Gene Ontology (GO) enrichment and Kyoto Encyclopedia of Genes and Genomes (KEGG) enrichment of genes upregulated in solute carrier family 7 member 5 (SLC7A5)‐high human samples. (C) Gene set enrichment analysis (GSEA) revealed the expression profiles of tryptophan catabolism‐related gene sets in both high and low expression groups of SLC7A5. (D) The clustering heatmap show the difference in the abundance of tryptophan metabolites between shNC and shSLC7A5 groups (*n* = 6). (E) Boxplots of XANA, Kyn and L‐tryptophan contents in shNC and shSLC7A5 cells (*n* = 6). (F) KEGG classification and enrichment plots of tryptophan metabolite abundance differences between shNC and shSLC7A5 cell lines (*n* = 6). (G) Isotope tracing with ^1^ ^3^C_11_‐tryptophan in shNC and shSLC7A5 RKO cells for 48 h. The relative abundance of ^1^ ^3^C_10_‐Kyn (M+10) and ^1^ ^3^C_10_‐XANA (M+10) was quantified by LC‒MS/MS after natural isotope abundance correction using accucor (*n* = 4). (H and I) The relative expression levels of tryptophan 2,3‐dioxygenase (TDO2), indoleamine‐2,3‐dioxygenase 1 (IDO1), AhR, CYP1A1 and CYP1B1 at different knockdown sites of SLC7A5 in RKO cells. (J) Expression of AhR‐responsive luciferase after treatment of RKO cells with specified concentrations of Kyn, XANA or a combination of both (*n* = 3). (K) Western blot analysis of AhR nuclear translocation in RKO cells following treatment with Kyn (50 µM) alone, XANA (50 µM) alone or their combination. (L) Confocal imaging of AhR nuclear translocation in RKO cells following treatment with Kyn (50 µM) alone, XANA (50 µM) alone or their combination (*n* = 3, scale bar = 100 µm). (M) SLC7A5‐knockdown RKO cells were treated with Kyn (50 µM), XANA (50 µM) or Kyn (50 µM) combined with XANA (50 µM), followed by EdU staining, plate clonality and apoptosis rate assays (*n* = 3, scale bar = 100 µm). (N) SLC7A5‐knockdown RKO cells were treated with AhR inhibitor CH‐223191 (10 µM) in combination with Kyn (50 µM), XANA (50 µM) or Kyn (50 µM) plus XANA (50 µM), followed by wound healing assays (*n* = 3, scale bar = 200 µm). (O) Western blot analysis was used to validate SLC7A5 overexpression and AhR‐knockout efficiency in RKO cells. (P) Colony formation and Transwell migration assays were used to assess the effect of CRISPR/Cas9‐mediated AhR knockout on the proliferation and migration of SLC7A5‐overexpressing RKO cells (*n* = 3, scale bar = 200 µm). Data are presented as mean ± SD, with individual data points shown. ^*^ *p* < .05, ^**^ *p* < .01, ^***^ *p* < .001.

To further investigate the mechanism of action of SLC7A5, we divided the TCGA dataset of CRC into high‐ and low‐expression groups. GO analysis revealed that SLC7A5‐high samples were enriched in amino acid processes, including L‐tryptophan catabolism to Kyn and amino acid transmembrane transporter activity (Figure 4A). Consistently, KEGG analysis and GSEA demonstrated the critical role of SLC7A5 in facilitating tryptophan uptake, transport and subsequent catabolic processes (Figure 4B,C). To identify tryptophan‐derived metabolites regulated by SLC7A5, we performed targeted metabolomics on shNC‐ and shSLC7A5‐transfected RKO cells and found that SLC7A5 knockdown significantly reduced the levels of XANA, Kyn and L‐tryptophan (Figure 4D,E). KEGG analysis demonstrated that these differing metabolites were predominantly associated with tryptophan metabolism, metabolic pathways and the biosynthesis of cofactors (Figure 4F). Isotope tracing with ^13^C_11_‐tryptophan confirmed that SLC7A5 knockdown significantly reduced the production of newly synthesised ^1^ ^3^C_10_‐Kyn (M+10) and ^1^ ^3^C_10_‐XANA (M+10), directly demonstrating that metabolic flux through the Kyn pathway is impaired despite compensatory indoleamine‐2,3‐dioxygenase 1 (IDO1) upregulation (Figure 4G). We further investigated the effects of SLC7A5 knockdown on the key tryptophan metabolic enzymes tryptophan 2,3‐dioxygenase (TDO2) and IDO1. The results revealed no significant difference in TDO2 expression after knockdown, whereas IDO1 expression was upregulated, which would not lead to a significant reduction in downstream metabolites, these data suggest that reduced tryptophan uptake may be the dominant factor contributing to decreased Kyn/XANA levels, despite compensatory IDO1 upregulation (Figure 4H). We subsequently selected metabolites with significant alterations identified through targeted metabolomics following SLC7A5 knockdown, Kyn and XANA, which are endogenous ligands of AhR^5^, ^6^ to further examine whether SLC7A5 facilitates CRLM via the Kyn/XANA‒AhR pathway. Western blot analysis revealed a significant decrease in the abundance of CYP1B1 and CYP1A1 after SLC7A5 knockdown, indicating reduced AhR activity (Figure 4I). Subsequently, we constructed RKO cells carrying the AhR luciferase reporter gene and reported that both Kyn and XANA could induce dose‐dependent AhR activation, with a synergistic effect observed when cells were treated in combination (Figure 4J). Western blotting revealed that Kyn and XANA could activate AhR and facilitate its nuclear translocation, most prominently in combination, thereby compensating for the diminished AhR activity resulting from SLC7A5 gene knockdown, which was further corroborated by fluorescence confocal microscopy, indicating that SLC7A5 exerts its effects through the Kyn/XANA‒AhR pathway (Figure 4K,L). Additionally, these exogenous tryptophan metabolites significantly restored the proliferation, migration and invasion of sh‐SLC7A5 RKO cells. However, the administration of the AhR antagonist CH‐223191 reversed these effects, as determined by wound healing and Transwell assays (Figures 4M,N and S4). To provide direct genetic evidence, we performed CRISPR/Cas9‐mediated AhR knockout in SLC7A5‐overexpressing RKO cells. SLC7A5 overexpression enhanced proliferation and migration, and this effect was reversed by AhR knockout (Figure 4O,P). Collectively, these findings indicate that SLC7A5 promotes CRC cell motility, invasiveness and metastatic dissemination via the Kyn/XANA–AhR signalling axis.

### 3.5 SLC7A5 knockdown reshapes the immune microenvironment of liver metastases

**FIGURE 5:**
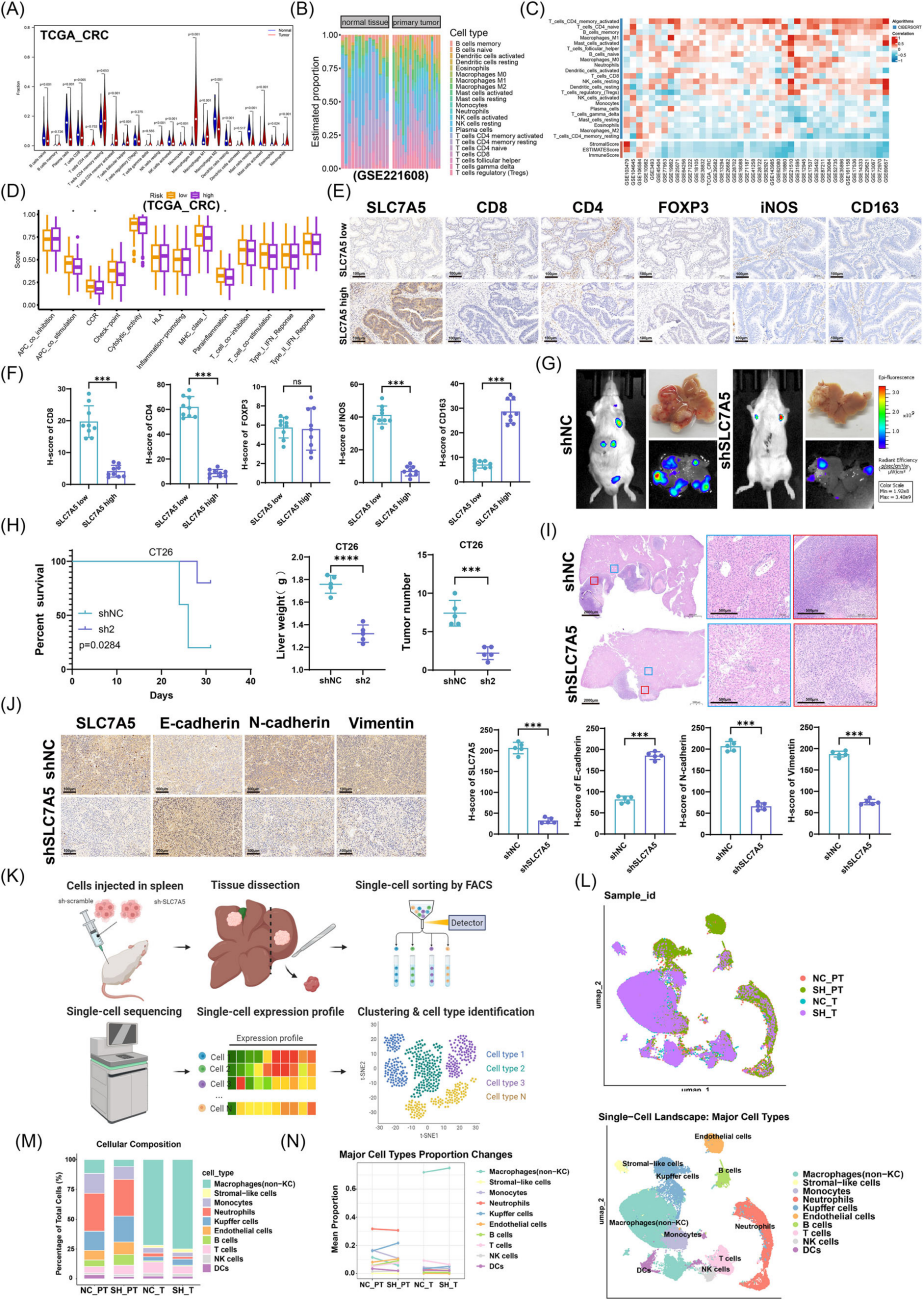
Solute carrier family 7 member 5 (SLC7A5) knockdown reshapes the immune microenvironment of liver metastases. (A and B) The violin plot and abundance stack plots of immune cell proportions demonstrated the proportional distribution of 22 immune cells in primary tumours and normal tissues. (C) Heatmaps of correlation analysis between target genes and immune cells. (D) The box plot of immune function showed the difference of immune function between the high expression group and the low expression group of SLC7A5. (E and F) Representative immunohistochemistry (IHC) images of CD8, CD4, FOXP3, iNOS and CD163 in tissues with high versus low SLC7A5 levels (*n* = 9, scale bar = 100 µm). (G and H) Representative bioluminescence images of colorectal liver metastasis (CRLM) models with corresponding overall survival, liver weights and percentage of metastatic lesions (*n* = 5). (I) Haematoxylin and eosin (H&E) staining of liver metastasis sections from CT26 cells in different groups (*n* = 5, scale bar = 2000 or 500 µm). (J) IHC analysis was used to evaluate the expression of E‐cadherin, N‐cadherin and Vimentin in CRLM model with different SLC7A5 levels (*n* = 5, scale bar = 100 µm). (K) CRLM models in the CT26 cells knocking down SLC7A5 or a vector control were used to examine cell subpopulations via single‐cell RNA sequencing (scRNA‐seq) (*n* = 5). (L) Uniform Manifold Approximation and Projection (UMAP) plot displays the predominant cell types in liver metastatic tumour tissues. (M and N) Stacked bars and line plots demonstrate the percentage composition and proportional changes of each cell type in liver metastatic tumour tissue. Data are presented as mean ± SD, with individual data points shown. ^*^ *p* < .05, ^**^ *p* < .01, ^***^ *p* < .001.

To further explore the association between SLC7A5 and the TIME, we stratified the TCGA‐CRC cohort into SLC7A5‐high and SLC7A5‐low subgroups. Significant differences in immune infiltration were observed between tumour and normal tissues, including altered proportions of naive B cells, CD8^+^ T cells, activated CD4^+^ memory T cells, and M0, M1 and M2 macrophages (Figure 5A,B). Notably, SLC7A5 expression showed a positive correlation with the enrichment of activated CD4^+^ memory T cells, naive CD4^+^ T cells, memory B cells and M1 macrophages (Figure 5C). The expression of antigen‐presenting cell (APC) costimulatory molecules and chemokine receptors was significantly reduced in the SLC7A5‐high group, suggesting that SLC7A5 overexpression may impair immune cell recruitment and antigen presentation, and lead to a loss of immune surveillance (Figure 5D). IHC staining confirmed that low SLC7A5 expression in CRC tissues was associated with high CD8, CD4 and iNOS levels and low CD163 levels, whereas high SLC7A5 expression was associated with the opposite pattern (Figure 5E,F). To investigate the impact of SLC7A5 on the TIME, we also conducted scRNA‐seq by establishing a CRLM model in BALB/c mice. Consistent with previous experimental results, knockdown of SLC7A5 reduced tumour bioluminescence, metastatic nodules, liver weight and EMT while prolonging survival (Figure 5G‒J). On day 30, liver samples were collected and categorised into four groups, and CD45^+^ cells were then isolated via flow cytometry for 10× Genomics scRNA‐seq (Figure 5K). After quality control and clustering (Figure S5A‒C), we identified major immune cell types (Figure S5D‒F), including macrophages (non‐Kupffer cells [KCs]), stromal‐like cells, monocytes, neutrophils and KCs, along with endothelial cells, B cells, T cells, natural killer cells and dendritic cells (DCs) (Figure 5L). Notably, SLC7A5 knockdown significantly altered the composition of immune cells, particularly the proportions of macrophages, T cells and B cells (Figure 5M,N).

### 3.6 Knockdown of SLC7A5 enhances macrophage antigen presentation and phagocytic capacity

**FIGURE 6:**
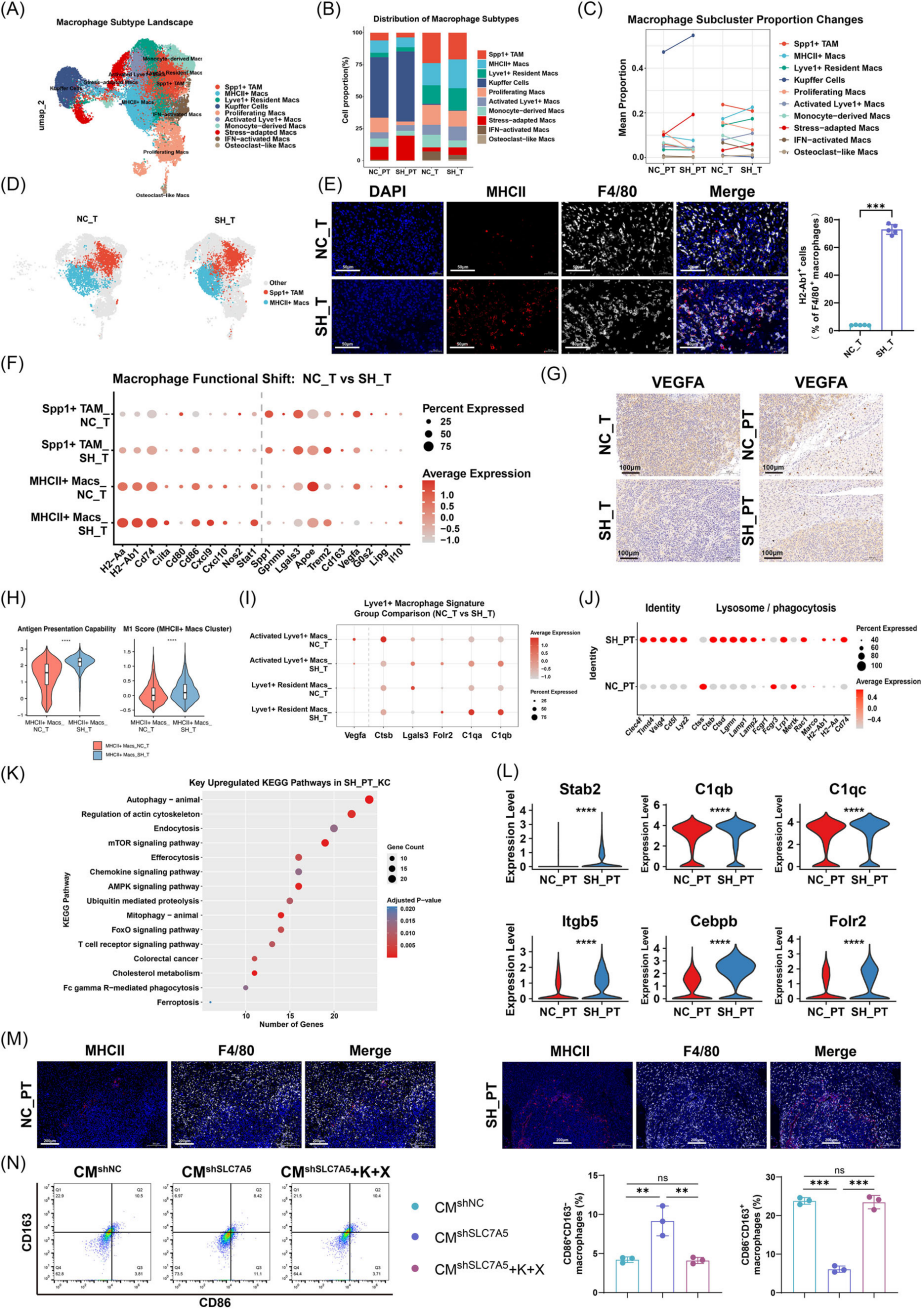
Knockdown of solute carrier family 7 member 5 (SLC7A5) enhances macrophage antigen presentation and phagocytic capacity. Uniform Manifold Approximation and Projection (UMAP) plot reveals the predominant macrophage subpopulations in liver metastatic tumour tissues. (B and C) Stacked bar and line graphs were used to display the percentage composition and proportional changes of major macrophage subpopulations in liver metastatic tumour tissues. (D) The UMAP plot demonstrates the distribution of Spp1^+^ tumour‐associated macrophages (TAMs) and MHCII^+^ Macs. (E) Multiplex immunohistochemistry (mIHC) demonstrated the changes in the proportion of MHCII^+^ Macs among total macrophages in the NC_T and SH_T groups (*n* = 5, scale bar = 50 µm). (F) The bubble heatmap displays the expression levels of Spp1^+^ TAM, MHCII^+^ Macs, and antigen‐presenting and immunosuppressive genes in tumour tissues. (G) Immunohistochemistry (IHC) analysis was used to evaluate the expression of VEGFA in colorectal liver metastasis (CRLM) model (*n* = 3, scale bar = 100 µm). (H) The violin plot shows the M1 score and antigen presentation ability of MHCII^+^ Macs in NC_T and SH_T groups. (I) The bubble heatmap displays representative gene expression profiles of Lyve1^+^ resident Macs and activated Lyve1^+^ Macs in the NC_T versus SH_T group. (J) The bubble heatmap shows the expression of typical marker genes for lysosome and phagocytosis in NC_PT versus SH_PT group. (K) Kyoto Encyclopedia of Genes and Genomes (KEGG) bubble diagram shows the main pathways of upregulated differential genes in the SH_PT group. (L) The violin plot shows the significantly upregulated differential genes in the SH_PT group burial pathway. (M) mIHC demonstrated the changes in the proportion of MHCII^+^ macrophages among total macrophages in the NC_PT and SH_PT groups (*n* = 5, scale bar = 200 µm). (N) Flow cytometry analysis was used to assess the effect of conditioned medium from SLC7A5‐knockdown CT26 cells on macrophage polarisation in bone marrow‐derived macrophages (BMDMs), with or without kynurenine (Kyn)/xanthurenic acid (XANA) supplementation (*n* = 3). Data are presented as mean ± SD, with individual data points shown. ^*^ *p* < .05, ^**^ *p* < .01, ^***^ *p* < .001.

Macrophages are key regulators within the TIME. Re‐clustering analysis of the macrophage compartment showed that KCs represented the dominant population in paracancerous tissues, while Spp1^+^ tumour‐associated macrophages (TAMs), MHCII^+^ macrophages and Lyve1^+^ resident macrophages were enriched in tumours and SLC7A5 knockdown reduced the proportions of Spp1^+^ TAMs (Figures 6A‒C and S6A‒D). Uniform Manifold Approximation and Projection (UMAP) and mIHC further revealed significant enrichment of MHCII^+^ macrophages in metastases upon SLC7A5 knockdown (Figure 6D,E). These MHCII^+^ macrophages exhibited increased expression of antigen presentation‐related genes (H2‐Aa, H2‐Ab1, Cd74, Ciita, Cd86, Cxcl10 and Stat1) and decreased expression of immunosuppressive markers (Apoe, Vegfa and IL‐10). Integrated M1 score and functional analyses confirmed that SLC7A5 knockdown enhanced the pro‐inflammatory and antigen‐presenting capacity of MHCII^+^ TAMs (Figure 6F‒H). We next focused on Lyve1‐expressing macrophage populations. In the NC_T group, increased VEGFA and CTSB suggested enhanced angiogenic and metastatic potential, whereas the SH_T group showed increased C1qa and C1qb, indicating improved antigen presentation and phagocytosis (Figure 6I). KCs constituted the largest proportion of the paracancerous tissue. In KCs adjacent to tumours, SLC7A5‐knockdown upregulated genes related to KCs identity (Clec4f, Timd4 and Vsig4), lysosomal function (Ctsb, Ctsd and Lamp1) and phagocytosis‐related pathways, such as endocytosis, efferocytosis (Stab 2, C1qb, C1qc and Itgb5) and Fc gamma R‐mediated phagocytosis, suggesting enhanced phagocytic and antitumour capacity (Figure 6J‒L). The mIHC results indicated that SLC7A5‐knockdown upregulated the proportion of macrophages expressing MHCII in paracancerous tissue, suggesting an improved capacity for antigen presentation (Figure 6M). To determine whether these macrophage phenotypic changes are driven by SLC7A5‐dependent metabolites, we performed conditioned medium experiments. Macrophages treated with conditioned medium from the SLC7A5‐knockdown group showed an increased proportion of CD86‐positive macrophages and a decreased proportion of CD163‐positive macrophages compared with those treated with conditioned medium from the shNC control group. When Kyn (50 µM) and XANA (50 µM) were added back to the conditioned medium from the SLC7A5‐knockdown group, the proportion of CD86‐positive macrophages decreased and the proportion of CD163‐positive macrophages increased, showing no significant difference from the shNC conditioned medium group (Figure 6N). Additionally, the increased expression of the chemokines Ccl9 and Ccl6 in the SH_T group indicated that SLC7A5 knockdown enhanced immune cell recruitment, the inflammatory response, and tissue repair capacity (Figure S6E).

### 3.7 Knockdown of SLC7A5 weakens the exhaustion phenotype of T cells and promotes their differentiation into Th1 or Th17 subsets

**FIGURE 7:**
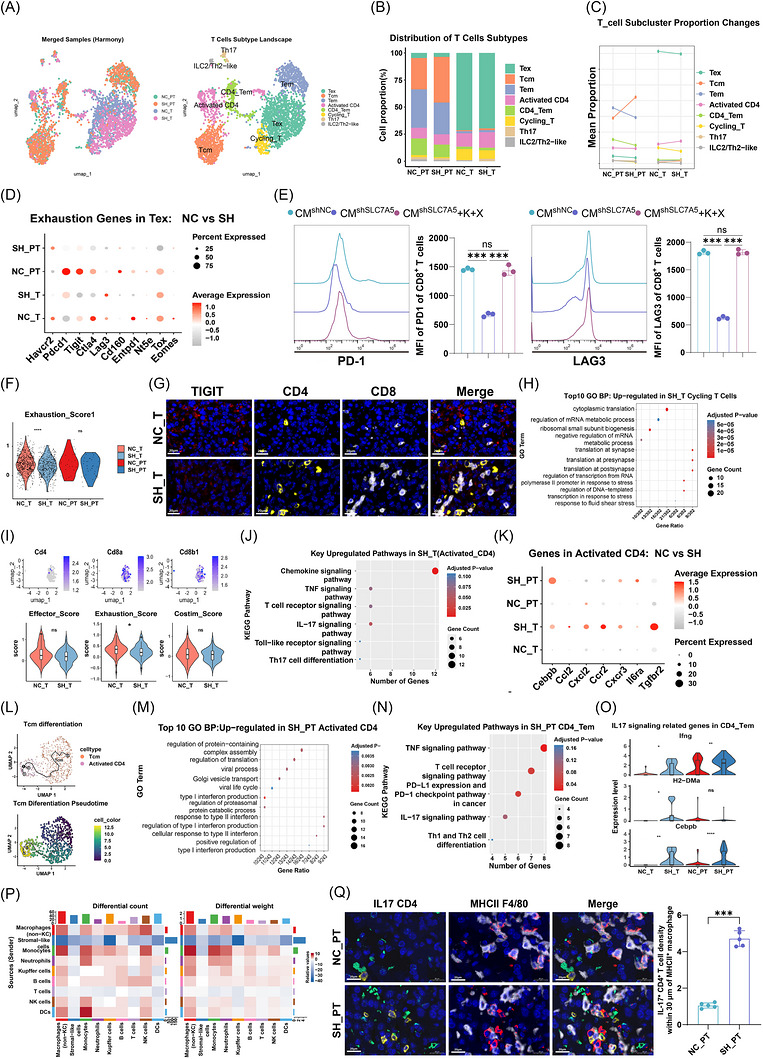
Knockdown of solute carrier family 7 member 5 (SLC7A5) weakens the exhaustion phenotype of T cells and promotes their differentiation into Th1 or Th17 subsets. (A) Uniform Manifold Approximation and Projection (UMAP) plot reveals the predominant T‐cell subsets in liver metastatic tumour tissues. (B and C) Stacked bar and line plots demonstrate the percentage composition and proportional changes of major T‐cell subsets in liver metastatic tumour tissues. (D) The bubble heatmap shows the expression of representative exhaustion genes in Tex. (E) Flow cytometry analysis was used to assess the effect of conditioned medium from SLC7A5‐knockdown CT26 cells on CD8^+^ T‐cell exhaustion markers in murine splenic CD8^+^ T cells, with or without kynurenine (Kyn)/xanthurenic acid (XANA) supplementation (*n* = 3). (F) The violin diagram shows the exhaustion score of four different groups. (G) Multiplex immunohistochemistry (mIHC) demonstrated TIGIT co‐localisation with CD4^+^ T cells and with CD8^+^ T cells in liver metastasis (*n* = 5, scale bar = 20 µm). (H) Gene Ontology (GO) analysis demonstrated the primary biological processes enriched in the differential gene upregulation of cycling T in the SH_T group. (I) The UMAP plot demonstrates the expression of representative genes associated with T‐cell function in cycling T cells. The violin plot shows the scores for killing, exhaustion and co‐stimulation of cycling T cells in the NC_T and SH_T groups. (J) Kyoto Encyclopedia of Genes and Genomes (KEGG) analysis reveals the primary pathways enriched in differential genes upregulated in SH_T group for activated CD4. (K) Bubble heatmap showed the main differential genes of activated CD4 in the four groups. (L) The pseudo‐time series analysis showed the differentiation trajectory of Tcm. (M) GO analysis shows the main biological processes of differential gene enrichment of upregulated activated CD4 in SH_PT group. (N) KEGG analysis illustrates the primary signalling pathways enriched in differentially upregulated genes of CD4 Tem in the SH_PT group. (O) The expression of IL‐17 signalling pathway‐related genes in the four groups. (P) The heatmap shows the differences in the quantity and intensity of cell communication between different immune cells in the SH_PT group. (Q) mIHC demonstrated the spatial distance between CD4^+^ T cells expressing IL‐17A and MHCII^+^ macrophages (*n* = 5, scale bar = 20 µm). Quantification of IL‐17^+^ CD4^+^ T‐cell density within 30 µm of MHCII^+^ macrophages in peritumoural tissues. SLC7A5 knockdown resulted in a significant increase in the density of IL‐17^+^ CD4^+^ T cells adjacent to MHCII^+^ macrophages in the SH_PT group compared with the NC_PT group (SH_PT: 4.72 ± .42 vs. NC_PT: 1.05 ± .14 cells/mm^2^, ^**^ *p* < .01). Data are presented as mean ± SD, with individual data points shown. ^*^ *p* < .05, ^**^ *p* < .01, ^***^ *p* < .001.

UMAP clustering revealed multiple CD8^+^ T‐cell subsets, including exhausted T cells (Tex), central memory T cells (Tcm) and effector memory T cells (Tem) (Figure 7A). Tex cells were the predominant subset within liver metastases, whereas Tcm and Tem cells were more abundant in paracancerous regions, suggesting that continuous antigen stimulation within the tumour drives CD8^+^ T cells towards exhaustion (Figure 7B,C). SLC7A5 knockdown reduced the transcript levels of exhaustion‐related genes, including Tigit, Ctla4 and Entpd1, in both metastatic and paracancerous liver tissues (Figure 7D). In conditioned medium experiments, conditioned medium from SLC7A5‐knockdown CT26 cells reduced the expression levels of PD‐1 and LAG3 on murine splenic CD8^+^ T cells, and this reduction was reversed when Kyn (50 µM) and XANA (50 µM) were added to the conditioned medium from the SLC7A5‐knockdown group (Figure 7E). Consistently, the exhaustion score remained lower in the SLC7A5‐knockdown group than in the NC_T group (Figure 7F). The results of mIHC also indicated a higher infiltration of CD4^+^ T cells and CD8^+^ T cells in SH_T group, along with decreased Tigit expression (Figure 7G). The cycling T subpopulation, which primarily comprises CD8+ T cells, exhibited increased replication, transcription activity in the SH_T group according to the results of the GO analysis (Figure 7H). Compared with the NC_T group, SH_T group also had a lower exhaustion score, suggesting that SLC7A5 knockdown may reverse the exhaustion of CD8^+^ T cells in tumours (Figure 7I). Activated CD4^+^ T cells, the second most abundant subset in tumours, were enriched in the knockdown group. GO and KEGG analyses revealed that SLC7A5‐knockdown enhanced T‐cell activation and differentiation, activated the IL‐17/tumor necrosis factor (TNF)/Th17 cell differentiation pathway, and upregulated the expression of Cebpb, Ccl2, Cxcl2, Ccr2, Cxcr3, Il6ra and Tgfbr2, suggesting that SLC7A5 knockdown promotes CD4^+^ T‐cell polarisation towards a cytotoxic Th17‐like phenotype with improved antitumour function (Figures 7J,K and S6F). Following SLC7A5 knockdown, Tcm infiltration increased in adjacent tumour tissues. Pseudotime analysis revealed that TCMs primarily differentiated into activated CD4^+^ T cells upon stimulation. GO enrichment revealed that upregulated genes in activated CD4^+^ T cells of SH_PT group were associated with type I and II interferon responses, suggesting a propensity towards CD4^+^ Th1 differentiation (Figure 7L,M). Unlike Tcm cells, Tem cells rapidly differentiate into effector cells upon antigen stimulation. KEGG analysis revealed that after SLC7A5 knockdown, the genes upregulated in CD4^+^ Tem cells were enriched in antigen processing and presentation and Th17 differentiation pathways in metastatic lesions, whereas in paracancerous tissue, they were enriched in the TNF, T cell receptor (TCR) and IL‐17 signalling pathways (Figures 7N,O and S6G). These findings suggest that SLC7A5 knockdown promotes CD4^+^ Tem differentiation towards a Th17 phenotype, enhancing IL‐17 signalling and antitumour efficacy. Cell‒cell communication analysis demonstrated a significant increase in both the number and intensity of inferred interactions within the SH_PT group relative to controls. mIHC further revealed significantly closer spatial proximity between IL‐17^+^ CD4^+^ T cells and MHCII^+^ macrophages in the SH_PT group, with a 4.5‐fold higher density of IL‐17^+^ CD4^+^ T cells within 30 µm of MHCII^+^ macrophages compared to the NC_PT group (Figure 7P,Q). SLC7A5 knockdown may thus alleviate immune suppression in adjacent tissues by activating intercellular signalling networks and enhancing local immune surveillance and inflammation.

### 3.8 SLC7A5 knockdown induces antigen‐driven and IgG‐secreting B‐cell clonal expansion in the TIME

**FIGURE 8:**
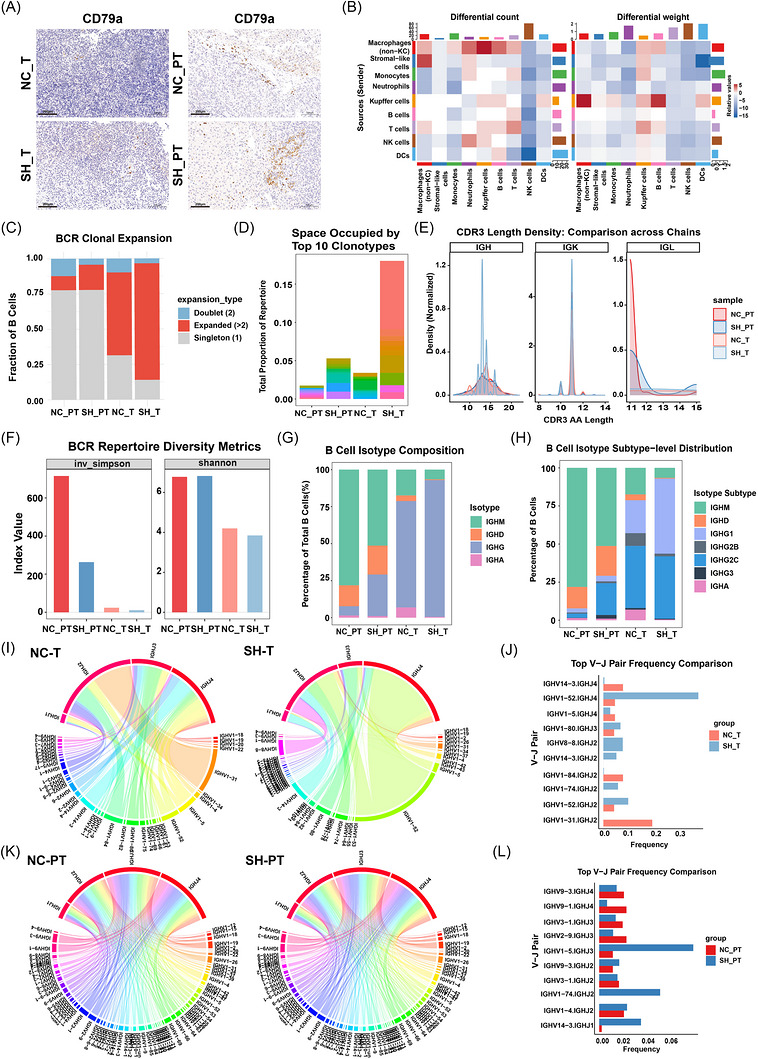
Knockdown of solute carrier family 7 member 5 (SLC7A5) induces antigen‐driven and immunoglobulin G (IgG)‐secreting B‐cell clonal expansion in the tumour immune microenvironment (TIME). (A) Representative staining for CD79a in NC‐T, SH_T, NC_PT and SH_PT groups (*n* = 5, scale bar = 200 µm). (B) The heatmap shows the differences in the quantity and intensity of cell communication, with a focus on the SH_T group compared to control group. (C) Stacked bar charts demonstrate the proportions of doublet, expanded and singleton clonal types across four groups. (D) The stacked bar chart displays the proportions of the top 10 most abundant BCR clonal types in the total immune repertoire across four groups. (E) The peak plot shows the normalised density of different complementary determining region 3 (CDR3) amino acid lengths in the three immunoglobulin chains. (F) The bar chart shows the variation in clonal diversity, as measured by the inverse Simpson and Shannon indices, across four groups. (G) The stacked bar chart displays the proportions of IGHM, IGHD, IGHG and IGHA across four groups. (H) The stacked bar chart shows the proportion of different subtypes of IGHG in the four groups. (I‒L) Circos plots and bar charts illustrate the frequency distribution of immunoglobulin V‐J gene pairings across the four groups. The most significantly altered V‐J pairs are highlighted. Data are presented as mean ± SD, with individual data points shown. ^*^ *p* < .05, ^**^ *p* < .01, ^***^ *p* < .001.

B cells serve as critical orchestrators of TIME remodelling, as their infiltration in paracancerous tissues is greater than that in metastases, with B‐cell enrichment upon SLC7A5 knockdown. Cell communication analysis revealed inactive cell interactions in the SH_T group, except for increased signalling from macrophages and KCs to B cells (Figure 8A,B). We subsequently examined the differences in the clonal characteristics of BCRs between the groups and demonstrated that SLC7A5 knockdown promoted robust antigen‐driven B‐cell clonal expansion within the TIME (Figure 8C,D). These expanded clones exhibited distinct CDR3 length characteristics, particularly within the immunoglobulin heavy chain (IGH) region (Figure 8E). The Shannon and inverse Simpson indices were reduced in tumour following SLC7A5 knockdown, indicating a more clonally focused B‐cell repertoire (Figure 8F). The marked reduction in the inverse Simpson within the PT group indicates that the peritumoural microenvironment is also subject to antigen‐driven clonal expansion of dominant B‐cell subsets, leading to reduced repertoire evenness and increased clonality. This suggests antigen‐driven expansion of dominant B‐cell clones within the TIME. Additionally, the proportion of immunoglobulin heavy chain gamma (IGHG), especially the IGHG1 subtype associated with antitumour immunity, increased following SLC7A5 knockdown, indicating enhanced B‐cell differentiation towards effector memory or IgG‐secreting cell phenotypes (Figure 8G,H). To investigate how the suppression of SLC7A5 promotes the expansion of antigen‐driven B‐cell clones, we analysed the characteristics of V/J gene pairing. SLC7A5 knockdown resulted in a significant skewing of V/J gene pair rearrangements in both tumour and adjacent tissues. Notably, the combination of IGHV1‐52 and IGHJ4 was particularly enriched within the tumour, indicating the presence of tumour‐associated predominant clones induced by SLC7A5 knockdown (Figure 8I‒L).

### 3.9 Association of SLC7A5 with therapeutic response in CRC

**FIGURE 9:**
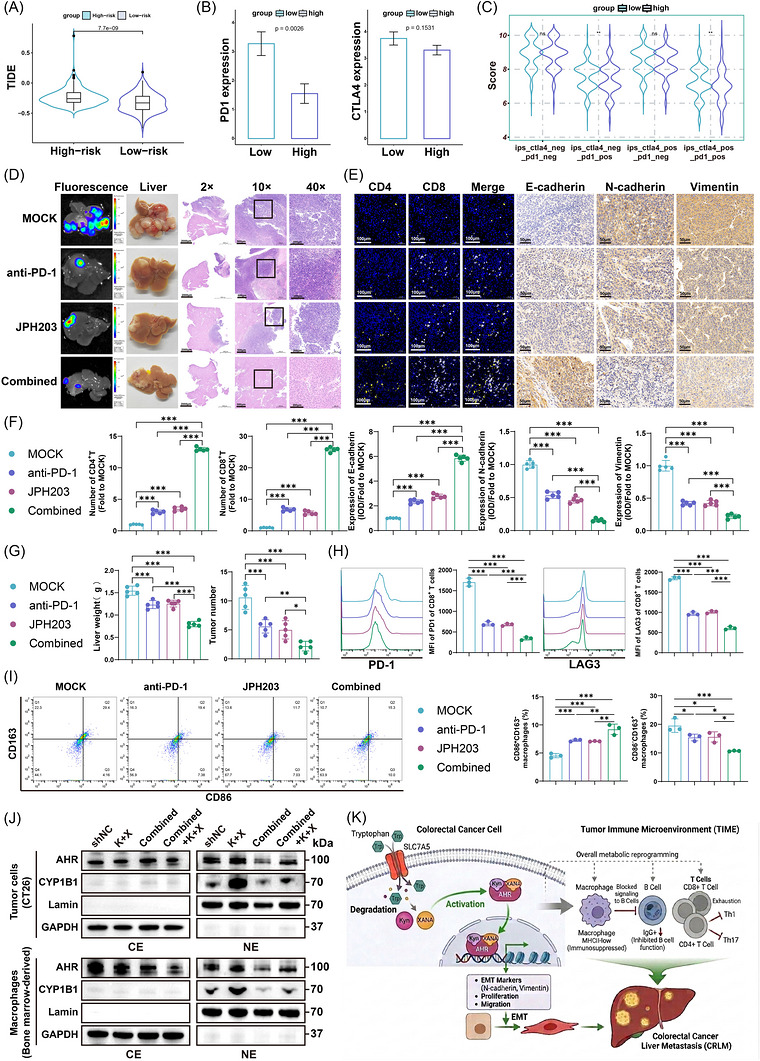
Association of solute carrier family 7 member 5 (SLC7A5) with therapeutic response in colorectal cancer (CRC). (A) Relative expression of TIDE in the high and low SLC7A5 groups. (B) Expression of PD1 and CTLA‐4 in patients with different SLC7A5 expression levels. (C) Response to PD1 and CTLA‐4 blockade immunotherapy in patients with different SLC7A5 expression levels. (D) Representative bioluminescence images and haematoxylin and eosin (H&E) staining images of liver tissues from the four groups (*n* = 5, scale bar = 2000 µm [overview], 500 µm [middle magnification] and 200 µm [high magnification]). (E and F) The levels of epithelial‒mesenchymal transition (EMT) markers, including E‐cadherin, N‐cadherin and Vimentin, and the degree of immune cell infiltration, including CD4^+^ T and CD8^+^ T cells, in the four groups (*n* = 5, scale bar = 100 or 50 µm). (G) Statistical analysis of metastatic nodules in liver sections in these four groups (*n* = 5). (H and I) Flow cytometry analysis was used to assess CD8^+^ T‐cell exhaustion markers (PD‐1 and LAG3) and macrophage polarisation markers (CD86 and CD163) in tumours from the four treatment groups (MOCK, anti‐PD‐1, JPH203 and combined) (*n* = 5). (J) Western blot analysis of CYP1B1 expression in CT26 cells and bone marrow‐derived macrophages (BMDMs) from the following groups: shNC, kynurenine (Kyn) (50 µM) plus xanthurenic acid (XANA) (50 µM), JPH203 (10 µM) plus anti‐PD‐1 (10 µg/mL), and JPH203 (10 µM) plus anti‐PD‐1 (10 µg/mL) with Kyn (50 µM) and XANA (50 µM). (K) Schematic representation showing the mechanism by which SLC7A5 promotes colorectal cancer liver metastasis by reprogramming tryptophan metabolism through the Kyn/XANA‒aryl hydrocarbon receptor (AhR) axis and reshaping the immune microenvironment. Data are presented as mean ± SD, with individual data points shown. ^*^ *p* < .05, ^**^ *p* < .01, ^***^ *p* < .001.

We subsequently used the Tumor Immune Dysfunction and Exclusion (TIDE) tool to assess the association between SLC7A5 and immune checkpoint inhibitor response in TCGA CRC samples. High SLC7A5 expression was linked to high TIDE scores, indicating poor immunotherapy response (Figure 9A). We further evaluated the relationship between SLC7A5 expression levels and the efficacy of immune checkpoint inhibitors. Low SLC7A5 expression was associated with high PD‐1 expression, whereas Cytotoxic T‐lymphocyte‐associated protein 4 (CTLA‐4) levels did not significantly differ between the groups (Figure 9B). In patients treated with anti‐PD‐1 alone or in combination with other therapies, the low‐SLC7A5 group had greater immune cell infiltration and a better treatment response, whereas anti‐CTLA‐4 monotherapy had no significant effect (Figure 9C). To confirm these observations, we employed a CRLM mouse model for subsequent in vivo studies. Mice were divided into four groups, namely vehicle, JPH203 (10 mg/kg), anti‐PD‐1 (10 µg/mouse) and combination, and administered the respective treatments intraperitoneally every 2 days for 28 days. Combination therapy significantly reduced tumour bioluminescence intensity, liver weight and metastatic nodule burden compared with either monotherapy, and exhibited increased infiltration of CD4^+^ and CD8^+^ T cells along with enhanced suppression of EMT (Figure 9D‒G). To further characterise the functional status of these infiltrating immune cells, we analysed tumours by flow cytometry. Combination therapy synergistically reduced CD8^+^ T‐cell exhaustion markers (PD‐1 and LAG3) and promoted macrophage pro‐inflammatory polarisation (CD86^+^CD163^−^) compared with monotherapy (Figure 9H,I). To investigate the molecular basis of this enhanced antitumour immunity, we performed in vitro experiments and found that combination treatment (JPH203 + anti‐PD‐1) suppressed AhR pathway activity in both CT26 cells and BMDMs, an effect reversed by Kyn/XANA supplementation (Figure 9J).

## 4 DISCUSSION

CRLM is a leading cause of mortality in patients with CRC. Investigating the mechanisms underlying its progression and identifying novel intervention targets have significant clinical implications. We found that SLC7A5 expression was significantly upregulated in CRLMs and primary tumours compared to normal tissues, an elevation that was tightly linked to poor survival outcomes in late‐stage patients. Mechanistically, SLC7A5 promotes the proliferation, invasion, metastasis and EMT of CRC cells by modulating the Kyn/XANA‒AhR axis. Additionally, scRNA‐seq revealed that SLC7A5 altered the local TIME. Silencing SLC7A5 induced macrophage polarisation towards the MHCII^+^ antigen‐presenting phenotype and enhanced their specific interactions with B cells. Furthermore, it effectively mitigated the exhaustion of CD8^+^ T cells and regulated the differentiation of CD4^+^ T cells into Th1/Th17 subsets. This study elucidates the dual metabolic‐immune mechanisms through which SLC7A5 facilitates CRLM and offers new prognostic markers and potential therapeutic targets for clinical applications.

While SLC7A5 overexpression has been documented across various malignancies and linked to poor prognosis,^7^, ^8^, ^9^ our findings extend beyond expression‐level correlations. We demonstrate a stepwise escalation of SLC7A5 from normal tissue through primary tumour to liver metastasis, with its prognostic significance specifically enriched in M1‐stage CRC, suggesting that SLC7A5 may function as a metastasis‐specific driver in CRC. In CRLM, SLC7A5 promotes metastatic outgrowth by mediating the reverse exchange of glutamine and extracellular leucine, thereby activating mTORC1 signalling and facilitating tumour cell adaptation to the liver microenvironment.^10^ This diversity of mechanisms implies that the pathological role of SLC7A5 is not solely determined by its expression level; it is also significantly influenced by the intrinsic genetic background of tumour cells and the metabolic characteristics of the microenvironment.

This study further elucidates the novel mechanism of SLC7A5 in CRLM. We discovered that SLC7A5 not only facilitates the uptake of essential amino acids but also activates the AhR signalling pathway by promoting the accumulation of metabolic products, such as Kyn and XANA, thereby driving the proliferation, metastasis and EMT of tumour cells. This mechanism is distinct from and complementary to previously reported roles of SLC7A5 in other malignancies or metabolic contexts. For instance, in triple‐negative breast cancer (TNBC), SLC7A5 enhances the glycolytic capacity of cancer cells by mediating the uptake of L‐tryptophan and activating the QPRT/NAD^+^ axis, thereby providing metabolic support for rapid proliferation.^11^ In CRC, MYC upregulates the expression of tryptophan transporters, including SLC7A5, to promote Kyn production, which sustains CRC cell proliferation.^12^ Furthermore, under conditions of metabolic stress, adaptive functional expansion of SLC7A5 occurs. For example, in the absence of arginine, it can function as a high‐affinity transporter of citrulline, allowing melanoma cells to circumvent nutritional deficiencies associated with arginine and survive. Under conditions of asparagine restriction, the activity of mTORC1 is preserved through the MAPK‒c‐MYC‒SLC7A5 axis, ensuring continuous cell proliferation in a nutrient‐deficient environment.^13^

The activation of AhR by tryptophan metabolites has emerged as a significant area of research at the intersection of tumour metabolism and immunity in recent years. Kyn, a crucial product of the tryptophan metabolic pathway, serves as a potent endogenous AhR agonist. Besides promoting the differentiation of regulatory T cells and tolerogenic myeloid cells, along with raising PD‐1 levels on CD8^+^ T lymphocytes, it also exerts a direct effect on the malignant properties of cancer cells.^14^ Research has indicated that in prostate cancer, the SLC7A5‒Kyn‒AhR signalling axis sustains the survival of dormant tumour cells induced by androgen deprivation therapy while also promoting their reactivation and recurrence progression, thereby contributing to treatment resistance.^15^ In addition to Kyn, its downstream metabolite, XANA, has been shown to exhibit agonistic activity against AhR. In TNBC, the endogenous accumulation of XANA within cells can continuously activate AhR, leading to the upregulation of TDO2 expression and the establishment of a positive feedback loop that accelerates tumour cell migration.^16^ This study further extends the role of SLC7A5 from a traditional ‘nutrient transporter’ to a ‘metabolic signal hub’, revealing a novel mechanism through which it drives malignant progression through the tryptophan metabolism‒AhR axis in CRC.

Notably, SLC7A5 complements, rather than overlaps with, the rate‐limiting enzymes IDO1 and TDO2 in its functional role. In our experiments, the upregulation of IDO1 upon SLC7A5 knockdown may represent a compensatory response to reduced intracellular tryptophan availability. However, this compensation is functionally insufficient—although IDO1 protein is elevated, isotope tracing confirmed that the production of newly synthesised Kyn and XANA remains significantly reduced, indicating that substrate availability, rather than enzymatic capacity, is the rate‐limiting step governing Kyn pathway flux. It does not directly engage in the metabolic transformation of tryptophan; instead, SLC7A5 facilitates the transmembrane uptake of neutral amino acids, including tryptophan, thereby supplying an adequate substrate for intracellular IDO1 and TDO2. This action indirectly enhances the metabolic flux of Kyn and amplifies the AhR activation signal. Although SLC7A5 and IDO1/TDO2 complement each other's functions, a synergistic amplification mechanism also exists between them. The supply of substrates from SLC7A5 enhances the metabolic transformation mediated by IDO1 and TDO2. This process results in increased production of Kyn, which is subsequently transaminated into kynurenic acid to stimulate AhR, thereby upregulating SLC7A5.^17^ This interaction establishes a positive regulatory loop of ‘uptake‐metabolism’ coupling, which perpetuates the malignant progression of tumours.

Understanding this mechanism has significant implications for clinical intervention strategies. Inhibitors that target the rate‐limiting enzyme IDO1 in upstream tryptophan metabolism have failed in phase III clinical trials because of the activation of the JAK2/STAT3 pathway within tumour cells, which facilitates immune escape.^18^ Moreover, the efficacy of pembrolizumab, which targets PD‐1, is limited in microsatellite‐stable CRC because of inadequate infiltration of effector T cells.^19^ These clinical challenges indicate that merely blocking the downstream catalytic steps of tryptophan metabolism or directly activating T cells is insufficient to effectively reverse the entrenched immunosuppressive TIME in CRC. In contrast, SLC7A5, which is regarded as the ‘upstream gateway’ for tryptophan uptake, offers distinct therapeutic advantages. Its highly selective inhibitor, JPH203, exhibited a favourable safety profile and promising clinical efficacy in phase I/II trials conducted for cholangiocarcinoma.^20^ In CRC, it inhibits EMT to resist tumour progression, with related phase II trials currently underway in populations with high SLC7A5 expression and RAS mutation.^21^ In addition to metabolic inhibition, JPH203 has been shown to possess immunomodulatory functions. In TNBC, it enhances anti‐PD‐1 therapy by downregulating PD‐L1 and promoting CD8^+^ T‐cell infiltration.^22^ In castration‐resistant prostate cancer, it inhibits CDK1/2 and mTOR/Akt signalling, thereby reversing resistance to cabazitaxel.^23^ These findings further establish SLC7A5 as a broad‐spectrum metabolic therapeutic target.

However, the clinical application of SLC7A5 inhibitors necessitates careful consideration of their dual role in different cell types. In tumour cells, SLC7A5 drives CRLM progression through the Kyn/XANA‒AhR pathway and promotes the formation of an immunosuppressive microenvironment. However, in CD8^+^ T cells, SLC7A5 is required for optimal effector function—it supports the cytotoxic activity of these cells by facilitating tryptophan uptake and subsequent activation of the AhR‒FasL axis.^24^ Therefore, systemic inhibition of SLC7A5 may suppress tumour growth and reduce tryptophan deprivation of CD8^+^ T cells, but at the same time it may impair the intrinsic tryptophan uptake capacity of CD8^+^ T cells, thereby compromising their antitumour activity. Future therapeutic strategies should aim to selectively target SLC7A5 in tumour cells while preserving its function in immune cells.

This study demonstrated that SLC7A5 knockdown drove macrophage repolarisation: the predominant Spp1^+^ immunosuppressive subset shifted to MHCII^+^ APCs, while KCs exhibited enhanced phagocytic and antigen‐presenting functions. Mechanistically, this repolarisation may result from SLC7A5 knockdown, which diminishes the production of the tryptophan metabolites Kyn and XANA. This reduction alleviated AhR‐mediated M2 polarisation^5^ redirecting macrophages from a pro‐tumour to an antitumour state.^14^, ^25^

Additionally, SLC7A5 knockdown promoted a exhaustion effect on CD8^+^ T cells and a shift in CD4^+^ T cells towards the Th1/Th17 phenotype. In contrast to PD‐1/PD‐L1 blockade, which directly activates T cells but often is ineffective in microsatellite‐stable CRCs because of inadequate T‐cell infiltration within tumours.^26^ SLC7A5 targets myeloid cells, such as TAMs, thereby indirectly activating T cells by alleviating upstream inhibition. After SLC7A5 knockdown, we observed a preference for specific V/J gene usage, such as IGHV1‐52 and IGHJ4, in IgG‐secreting B cells within metastatic lesions. Previous studies have demonstrated that IgA‐secreting B cells inhibit CD8^+^ T cells, thereby facilitating tumour evasion of immune attack.^27^ This study confirmed the expansion of IgG‐secreting B cells rather than IgA‐secreting B cells, which aligns with the composition of B cells within tertiary lymphoid structures in melanoma and sarcoma and is often indicative of a favourable response to immunotherapy.^28^ In contrast to the typical polyclonal amplification observed in general inflammation, this restricted BCR lineage suggests a robust, antigen‐driven adaptive immune response.^29^ These results, together with CellChat analyses, indicated that following SLC7A5 inhibition, enhanced signalling from MHCII^+^ macrophages and B cells improved antigen presentation. This drove the expansion of specific IgG‐secreting B‐cell clones and amplified antitumour immunity through antibody‐dependent cell‐mediated cytotoxicity and further antigen presentation to T cells.^28^ Ultimately, this process leads to a self‐reinforcing immune activation mechanism, which is consistent with the function of the tertiary lymphoid structure.^30^

At the molecular level, knockdown of the SLC7A5 gene specifically depletes Folr2^+^ macrophages and promotes effector T‐cell infiltration and activation. The level of Folr2, a validated indicator of tissue‐resident macrophages responsible for maintaining immune tolerance^31^, ^32^ is downregulated, further confirming the disruption of the immunosuppressive TIME.^33^ This observation aligns with findings in primary liver cancer, where FOLR2^+^ TAMs can form an endothelial barrier that rejects CD8^+^ T cells.^34^ Our data extend this mechanism from primary liver cancer to metastatic environments, suggesting that the exhaustion of Folr2^+^ macrophage subsets due to SLC7A5 knockdown may be a critical factor promoting effector T‐cell infiltration and activation.

However, several important limitations of this work should be recognised. First, scRNA‐seq data were generated from a single experimental batch; independent cohort validation is warranted. Second, in vivo validation using AhR‐deficient CRC cells in the intrasplenic CRLM model was not performed. Third, IDO1 overexpression experiments in SLC7A5‐deficient cells, which would provide complementary evidence by testing whether enhancing enzymatic capacity can rescue the metastatic phenotype, were not performed and represent a direction for future investigation. Fourth, while CM experiments demonstrated that SLC7A5‐derived metabolites directly regulate macrophage polarisation and T‐cell exhaustion, CM experiments assessing B‐cell differentiation and IgG secretion were not performed, and functional validation of B‐cell responses through ADCC assays with purified IgG and in vivo B‐cell depletion experiments is needed to establish whether these B cells are functionally required for the enhanced antitumour immunity. Fifth, while our CM experiments demonstrate that tumour‐derived soluble factors directly regulate macrophage and T‐cell function, Transwell co‐culture experiments comparing the effects of soluble factors versus direct cell‒cell contact were not performed; such studies would further distinguish the relative contributions of these two modes of immune regulation. Sixth, CellChat analyses represent computational predictions; the inferred intercellular interactions require future validation using independent cohorts and functional assays, such as ligand‒receptor blockade experiments or spatial transcriptomic approaches.

In summary, our findings identify SLC7A5 as a key metabolic gatekeeper that drives CRC cell proliferation and metastasis by signalling through the Kyn/XANA‒AhR axis. Inhibiting SLC7A5 initiates a cascading failure of the TIME. Targeting SLC7A5 not only deprives tumours of vital nutrients but also concurrently activates the TIME. These findings establish a robust foundation for the combined use of metabolic inhibitors and immunotherapies to address drug resistance in patients with CRLM.

## AUTHOR CONTRIBUTIONS

Junming Xu conceived and supervised the study. Yaohao Luo, Lei Li and Shanbao Li designed and performed study. Xinshuai Wang assisted with cell experiments. Zeping He contributed in bioinformatics analysis. Fangbin Song, Jun Qin and Jinyan Zhang helped with specimen acquirement and manuscript revision. All the authors read and approved the final manuscript.

## CONFLICT OF INTEREST STATEMENT

The authors declare they have no conflicts of interest.

## ETHICS STATEMENT

The study involving human participants was reviewed and approved by the Institutional Ethics Committee of Shanghai General Hospital (approval no. 2024SQ521). All participants provided written informed consent prior to enrolment. The animal experiments were conducted in accordance with institutional guidelines and were approved by the Institutional Animal Care and Use Committee of Shanghai General Hospital (approval no. 2025AW008). All procedures followed the relevant national and institutional guidelines for the care and use of laboratory animals.

## Supporting information



Supporting Information

## Data Availability

The single‐cell RNA sequencing data generated in this study have been deposited in the NCBI Sequence Read Archive (SRA) under BioProject accession PRJNA1495808. The targeted metabolomics data have been deposited in MetaboLights under study accession MTBLS15074. Any additional data supporting the findings of this study are available from the corresponding author upon reasonable request.

## References

[1] Koimtzis G , Geropoulos G , Stefanopoulos L , et al. The role of carbon nanoparticles as lymph node tracers in colorectal cancer: a systematic review and meta‐analysis. Int J Mol Sci. 2023;24(20):15293. 37894972 10.3390/ijms242015293 PMC10607187

[2] Liu S , Zhang Z , Wang Z , et al. SPP1 drives colorectal cancer liver metastasis and immunotherapy resistance by stimulating CXCL12 production in cancer‐associated fibroblasts. Cancer Res. 2026;86(1):58‐79. 41051794 10.1158/0008-5472.CAN-24-4916 PMC12757724

[3] Zhou M , Guan B , Liu Y , et al. Fibrinogen‐like 2 in tumor‐associated macrophage‐derived extracellular vesicles shapes an immunosuppressive microenvironment in colorectal liver metastases by promoting tumor stemness and neutrophil extracellular traps formation. Cancer Lett. 2025;618:217642. 40097065 10.1016/j.canlet.2025.217642

[4] Väyrynen V , Wirta EV , Seppälä T , et al. Incidence and management of patients with colorectal cancer and synchronous and metachronous colorectal metastases: a population‐based study. BJS Open. 2020;4(4):685‐692. 32543788 10.1002/bjs5.50299 PMC7397359

[5] Opitz CA , Litzenburger UM , Sahm F , et al. An endogenous tumor‐promoting ligand of the human aryl hydrocarbon receptor. Nature. 2011;478(7368):197‐203. 21976023 10.1038/nature10491

[6] Michaudel C , Danne C , Agus A , et al. Rewiring the altered tryptophan metabolism as a novel therapeutic strategy in inflammatory bowel diseases. Gut. 2023;72(7):1296‐1307. 36270778 10.1136/gutjnl-2022-327337 PMC10314090

[7] Duan S , Li X , Song C , et al. Isoliquiritigenin inhibits triple‐negative breast cancer progression via targeting the IRF5/SLC7A5/IDO1‐mediated tryptophan metabolism pathway. Oncol Res. 2025;33(11):3543‐3556. 41179282 10.32604/or.2025.068292 PMC12573168

[8] Lin YJ , Wu YC , Liu YJ , et al. Targeting SLC7A5 in lung squamous cell carcinoma: implications for cancer metabolism shift and boron neutron capture therapy resistance. Oncogenesis. 2025;14(1):26. 40701982 10.1038/s41389-025-00568-z PMC12287270

[9] Huang R , Wang H , Hong J , et al. Targeting glutamine metabolic reprogramming of SLC7A5 enhances the efficacy of anti‐PD‐1 in triple‐negative breast cancer. Front Immunol. 2023;14:1251643. 37731509 10.3389/fimmu.2023.1251643 PMC10507177

[10] Najumudeen AK , Ceteci F , Fey SK , et al. The amino acid transporter SLC7A5 is required for efficient growth of KRAS‐mutant colorectal cancer. Nat Genet. 2021;53(1):16‐26. 33414552 10.1038/s41588-020-00753-3

[11] Fedoroff MY , Zhao L , Wang S , et al. Amino acid transporter LAT1 (SLC7A5) promotes metabolic rewiring in TNBC progression through the L‐Trp/QPRT/NAD(+) pathway. J Exp Clin Cancer Res. 2025;44(1):190. 40611146 10.1186/s13046-025-03446-z PMC12224598

[12] Venkateswaran N , Conacci‐Sorrell M . Kynurenine: an oncometabolite in colon cancer. Cell Stress. 2020;4(1):24‐26. 31922097 10.15698/cst2020.01.210 PMC6946015

[13] Pathria G , Verma S , Yin J , Scott DA , Ronai ZA . MAPK signaling regulates c‐MYC for melanoma cell adaptation to asparagine restriction. EMBO Rep. 2021;22(3):e51436. 33554439 10.15252/embr.202051436 PMC7926261

[14] Campesato LF , Budhu S , Tchaicha J , et al. Blockade of the AHR restricts a Treg‐macrophage suppressive axis induced by L‐Kynurenine. Nat Commun. 2020;11(1):4011. 32782249 10.1038/s41467-020-17750-z PMC7419300

[15] Li S , Zhang Y , Luo M , et al. AR to GR switch modulates differential TDO2‒Kyn‒AhR signaling to promote the survival and recurrence of treatment‐induced dormant cells in prostate cancer. Cell Discov. 2025;11(1):67. 40759641 10.1038/s41421-025-00817-w PMC12322048

[16] Novikov O , Wang Z , Stanford EA , et al. An aryl hydrocarbon receptor‐mediated amplification loop that enforces cell migration in ER‒/PR‒/Her2‒ human breast cancer cells. Mol Pharmacol. 2016;90(5):674‐688. 27573671 10.1124/mol.116.105361 PMC5074452

[17] Badawy AA . Tryptophan metabolism and disposition in cancer biology and immunotherapy. Biosci Rep. 2022;42(11):BSR20221682. 36286592 10.1042/BSR20221682 PMC9653095

[18] Yu L , Xu L , Chen Y , et al. IDO1 inhibition promotes activation of tumor‐intrinsic STAT3 pathway and induces adverse tumor‐protective effects. J Immunol. 2024;212(7):1232‐1243. 38391297 10.4049/jimmunol.2300545

[19] Yamaguchi K , Tsuchihashi K , Ueno S , et al. Efficacy of pembrolizumab in microsatellite‐stable, tumor mutational burden‐high metastatic colorectal cancer: genomic signatures and clinical outcomes. ESMO Open. 2025;10(1):104108. 39765187 10.1016/j.esmoop.2024.104108 PMC11758824

[20] Okano N , Naruge D , Kawai K , et al. First‐in‐human phase I study of JPH203, an L‐type amino acid transporter 1 inhibitor, in patients with advanced solid tumors. Invest New Drugs. 2020;38(5):1495‐1506. 32198649 10.1007/s10637-020-00924-3

[21] Otani R , Takigawa H , Yuge R , et al. The anti‐tumor effect of the newly developed LAT1 inhibitor JPH203 in colorectal carcinoma, according to a comprehensive analysis. Cancers (Basel). 2023;15(5):1383. 36900176 10.3390/cancers15051383 PMC10000236

[22] Zhao Y , Pu C , Liu K , Liu Z . Targeting LAT1 with JPH203 to reduce TNBC proliferation and reshape suppressive immune microenvironment by blocking essential amino acid uptake. Amino Acids. 2025;57(1):27. 40379991 10.1007/s00726-025-03456-3 PMC12084285

[23] Rii J , Sakamoto S , Mizokami A , et al. L‐type amino acid transporter 1 inhibitor JPH203 prevents the growth of cabazitaxel‐resistant prostate cancer by inhibiting cyclin‐dependent kinase activity. Cancer Sci. 2024;115(3):937‐953. 38186218 10.1111/cas.16062 PMC10920979

[24] Lu C , Liang L , Wu Y , et al. Slc7a5 promotes T cell anti‐tumor immunity through sustaining cytotoxic T lymphocyte effector function. Oncogene. 2025;44(41):3939‐3954. 40858813 10.1038/s41388-025-03543-5 PMC12560258

[25] Mezrich JD , Fechner JH , Zhang X , Johnson BP , Burlingham WJ , Bradfield CA . An interaction between kynurenine and the aryl hydrocarbon receptor can generate regulatory T cells. J Immunol. 2010;185(6):3190‐3198. 20720200 10.4049/jimmunol.0903670 PMC2952546

[26] Le DT , Uram JN , Wang H , et al. PD‐1 blockade in tumors with mismatch‐repair deficiency. N Engl J Med. 2015;372(26):2509‐2520. 26028255 10.1056/NEJMoa1500596 PMC4481136

[27] Shalapour S , Lin XJ , Bastian IN , et al. Inflammation‐induced IgA^+^ cells dismantle anti‐liver cancer immunity. Nature. 2017;551(7680):340‐345. 29144460 10.1038/nature24302 PMC5884449

[28] Cabrita R , Lauss M , Sanna A , et al. Tertiary lymphoid structures improve immunotherapy and survival in melanoma. Nature. 2020;577(7791):561‐565. 31942071 10.1038/s41586-019-1914-8

[29] Helmink BA , Reddy SM , Gao J , et al. B cells and tertiary lymphoid structures promote immunotherapy response. Nature. 2020;577(7791):549‐555. 31942075 10.1038/s41586-019-1922-8 PMC8762581

[30] Petitprez F , de Reyniès A , Keung EZ , et al. B cells are associated with survival and immunotherapy response in sarcoma. Nature. 2020;577(7791):556‐560. 31942077 10.1038/s41586-019-1906-8

[31] Ramachandran P , Dobie R , Wilson‐Kanamori JR , et al. Resolving the fibrotic niche of human liver cirrhosis at single‐cell level. Nature. 2019;575(7783):512‐518. 31597160 10.1038/s41586-019-1631-3 PMC6876711

[32] Puig‐Kröger A , Sierra‐Filardi E , Domínguez‐Soto A , et al. Folate receptor beta is expressed by tumor‐associated macrophages and constitutes a marker for M2 anti‐inflammatory/regulatory macrophages. Cancer Res. 2009;69(24):9395‐9403. 19951991 10.1158/0008-5472.CAN-09-2050

[33] Ramos RN , Missolo‐Koussou Y , Gerber‐Ferder Y , et al. Tissue‐resident FOLR2(+) macrophages associate with CD8(+) T cell infiltration in human breast cancer. Cell. 2022;185(7):1189‐1207.e25. 35325594 10.1016/j.cell.2022.02.021

[34] Sharma A , Seow JJW , Dutertre CA , et al. Onco‐fetal reprogramming of endothelial cells drives immunosuppressive macrophages in hepatocellular carcinoma. Cell. 2020;183(2):377‐394.e21. 32976798 10.1016/j.cell.2020.08.040

