# Supplementary material for: SLC7A5 promotes colorectal cancer liver metastasis by reprogramming tryptophan metabolism through the Kyn/XANA‒AhR axis and reshaping the immune microenvironment
Source: Clin Transl Med. 2026 Aug 6;16(8):e70766. doi: 10.1002/ctm2.70766 (PMC13448136; doi:10.1002/ctm2.70766)
Supplement: Supplementary file 1 [file CTM2-16-e70766-s001.docx]

**Supplementary Material for “SLC7A5 promotes colorectal cancer liver metastasis by reprogramming tryptophan metabolism through the Kyn/XANA-AhR axis and reshaping the immune microenvironment”**

Yaohao Luo^1*^, Lei Li^1*^, Shanbao Li^1*^, Xinshuai Wang^1*^, Zeping He^1^, Fangbin Song^1^, Jun Qin^1^, Jinyan Zhang^1#^, Junming Xu^1#^

^1^Department of General Surgery, Shanghai General Hospital, Shanghai Jiao Tong University School of Medicine, Shanghai 200080, China

*Y.L., L.L., L.S. and X.W. contributed equally to this study

^#^Author to whom correspondence should be addressed: 000183@sjtu.edu.cn

**Contents in Supplementary File**

1、Supplementary Materials and Methods

2、Supplementary Figure S1-6 and Legends

3、Supplementary Table S1-3

**1、Supplementary Materials and Methods**

**Quantitative reverse transcription-polymerase chain reaction (qRT-PCR)**

RNA was extracted from cells using TRIzol reagent (Takara, Japan) and then reverse transcribed into cDNA with PrimeScript RT Master Mix (EnzyArtisan, China). qRT-PCR was conducted using SYBR Green (EnzyArtisan, China), and the expression of target genes was normalized to that of GAPDH. Primer sequences are provided in Table S1.

**Western blot**

Total protein was extracted from cells or tissues using RIPA buffer containing 1% PMSF. Nuclear and cytoplasmic fractions were isolated with a protein extraction kit (Thermo Scientific, USA). Protein lysates were resolved by SDS-PAGE and transferred onto PVDF membranes (Millipore, USA). Membranes were blocked with 5% skim milk and incubated sequentially with primary and secondary antibodies. Blots were developed using ECL reagent (Absin, China) and quantified with ImageJ. Antibody details are provided in Table S2.

**Pharmacological treatments**

For SLC7A5 inhibition, cells were treated with 25 μM and 50 μM BCH (MedChemExpress, China) as previously described^1^. Rescue experiments were performed by adding 50 μM Kyn (MedChemExpress, China)^2^ and 50 μM XANA (MedChemExpress, China)^3^ with or without 10 μM CH-223191 (MedChemExpress, China)^4^. For combination therapy in vitro, cells were treated with 10 μM JPH203 (Sparkjade, China)^5^ and 10 μg/mL anti-PD-1 antibody (clone RMP1-14, Bio X Cell, USA)^6^. For the in vivo combination therapy model, mice received intraperitoneal injections of 10 μg anti-PD-1 antibody (clone RMP1-14, Bio X Cell, USA)^7^ and JPH203 at 10 mg/kg (Sparkjade, China)^8^ every 2 days for 28 days.

**CCK-8 assay**

For the CCK-8 assay, cells were plated into 96-well plates (3,000 cells/well in 100 μL complete medium). At the indicated time points, 10 μL CCK-8 reagent (NCM Biotech, China) was added, and plates were incubated at 37°C for 2 h before measuring the absorbance at 450 nm.

**Colony formation assay**

Cells were seeded in 6-well plates at 1,000 cells per well. After 14 days, colonies were fixed with 4% paraformaldehyde for 30 min, stained with 1% crystal violet for 20 min, and counted.

**EdU proliferation assay**

For EdU proliferation assay, cells were seeded into 96-well plates and subsequently Cell-Light EdU Apollo 567 (RiboBio, China) was performed for 2h to mark proliferating cells.

**Cell apoptosis**

Cells were cultured to 70%-80% confluence and treated with apoptosis-inducing compounds A (Beyotime-C0006S, China) and B (Beyotime-C0005, China) for 12 h. Cells were then collected, stained with Annexin V-APC and PI for 15 min at room temperature, and analyzed by flow cytometry.

**Transwell assay**

Cell migration and invasion were assessed using Transwell chambers with or without Matrigel coating. RKO and CT26 cells were resuspended in 200 μL of serum-free medium and seeded into the upper chamber. The lower chamber was filled with 500 μL of complete medium containing 10% FBS as a chemoattractant. After incubation for 72 h, cells remaining on the upper surface of the membrane were removed with a cotton swab, while cells that had migrated or invaded through the membrane were fixed, stained with crystal violet, and counted under a light microscope.

**Wound healing assay**

Cells were seeded in 6-well plates and grown to near confluence. The monolayer was serum-starved for 24 h and then scratched with a sterile 200 μL pipette tip to create a linear wound. After scratching, cells were kept in serum-free medium, and wound healing was photographed at 0 h and 72 h post-wounding. Wound closure was quantified to assess migration rate.

**References**

1.	Ohshima Y, Kaira K, Yamaguchi A, Oriuchi N, Tominaga H, Nagamori S, et al. Efficacy of system l amino acid transporter 1 inhibition as a therapeutic target in esophageal squamous cell carcinoma. Cancer Sci. 2016;107(10):1499-505.

2.	Stier MT, Sewell AE, Mwizerwa EL, Sim CY, Tanner SM, Nichols CM, et al. Metabolic adaptations rewire CD4(+) T cells in a subset-specific manner in human critical illness with and without sepsis. Nat Immunol. 2026;27(2):236-49.

3.	Copeland CS, Neale SA, Salt TE. Actions of Xanthurenic acid, a putative endogenous Group II metabotropic glutamate receptor agonist, on sensory transmission in the thalamus. Neuropharmacology. 2013;66:133-42.

4.	Kim SH, Henry EC, Kim DK, Kim YH, Shin KJ, Han MS, et al. Novel compound 2-methyl-2H-pyrazole-3-carboxylic acid (2-methyl-4-o-tolylazo-phenyl)-amide (CH-223191) prevents 2,3,7,8-TCDD-induced toxicity by antagonizing the aryl hydrocarbon receptor. Mol Pharmacol. 2006;69(6):1871-8.

5.	Yun DW, Lee SA, Park MG, Kim JS, Yu SK, Park MR, et al. JPH203, an L-type amino acid transporter 1-selective compound, induces apoptosis of YD-38 human oral cancer cells. J Pharmacol Sci. 2014;124(2):208-17.

6.	Zander RA, Obeng-Adjei N, Guthmiller JJ, Kulu DI, Li J, Ongoiba A, et al. PD-1 Co-inhibitory and OX40 Co-stimulatory Crosstalk Regulates Helper T Cell Differentiation and Anti-Plasmodium Humoral Immunity. Cell Host Microbe. 2015;17(5):628-41.

7.	Moynihan KD, Opel CF, Szeto GL, Tzeng A, Zhu EF, Engreitz JM, et al. Eradication of large established tumors in mice by combination immunotherapy that engages innate and adaptive immune responses. Nat Med. 2016;22(12):1402-10.

8.	Wempe MF, Rice PJ, Lightner JW, Jutabha P, Hayashi M, Anzai N, et al. Metabolism and pharmacokinetic studies of JPH203, an L-amino acid transporter 1 (LAT1) selective compound. Drug Metab Pharmacokinet. 2012;27(1):155-61.

**2、Supplementary Figure S1-6 and Legends**

**Figure. S1**

**Figure. S1**: Kaplan-Meier survival curves stratified by SLC7A5 expression levels in those with stage III–IV (compared to stage I–II), T4 (compared to T2–T3), and N1-N2 (compared to N0) disease.

**Figure. S2**

**Figure. S2**: (A-C) CCK-8, Colony formation and EdU assay was used to evaluate the proliferative capacity of SLC7A5 knockdown or inhibition groups in CT26 cells (n=3, Scale bar = 100 µm). Data are presented as mean ± SD, with individual data points shown. * P < 0.05, ** P < 0.01, *** P < 0.001

**Figure. S3**

**Figure. S3**: (A) Representative bioluminescence images of the Livers and lungs in the CRLM model (n=5). (B) H-score of EMT-related proteins in patient samples with low or high SLC7A5 expression (n=9). (C) Western blot analyses were performed to assess the expression of Snail and Slug in RKO cells. Data are presented as mean ± SD, with individual data points shown. * P < 0.05, ** P < 0.01, *** P < 0.001

**Figure. S4**

**Figure. S4**: SH-SLC7A5 RKO cells were treated with AhR inhibitor CH-223191 (10μM) in combination with Kyn (50μM), XANA (50μM), or Kyn (50 μM) plus XANA (50μM), followed by transwell assays (n=3, Scale bar = 200 µm). Data are presented as mean ± SD, with individual data points shown. * P < 0.05, ** P < 0.01, *** P < 0.001

**Figure. S5**

**Figure. S5**: (A) The PC_Elbowplot illustrates the standard deviation explained by each principal component. (B) Quality control (QC) and screening of raw data, performing subset screening to filter out fragments, doublets, and dead/defective cells. (C) UAMP plots after QC and doublet removal. (D) Heat maps demonstrate the expression of the five most significantly altered major representative genes for each cell family across the four groups. (E) Heat maps of bubble plots show the two most significantly altered markers for each cell family. (F) The bubble heat map illustrates the distribution differences of the most significantly altered markers corresponding to each cell family across four distinct groups.

**Figure. S6**

**Figure. S6**: (A) The Mac_Elbowplot displays the standard deviation explained by each principal component. (B) UMAP plot of macrophage subsets. (C) Bubble plot showing expression of representative markers in different macrophage subsets. (D) UMAP plots after removing the three mixed subgroups. (E) Violin plot showing the relative level comparison of cytokines and chemokines in the differential gene analysis results. (F) GO bubble plot illustrating the major biological processes enriched in the upregulated differential genes of Activated CD4 in the SH_T group. (G) KEGG bubble plot demonstrating the major signaling pathways enriched in the upregulated differential genes of CD4 Tem in the SH_T group.

**3、Supplementary Table S1-3**

**Table. S1 Primers used for qRT–PCR in this study**

| **Gene** | **Primer-F** | **Primer-R** |
| --- | --- | --- |
| Human-SLC7A5 | CCGTGAACTGCTACAGCGT | CTTCCCGATCTGGACGAAGC |
| Mouse-SLC7A5 | GGTCTCTGTTCACGTCCTCAAG | GAACACCAGTGATGGCACAGGT |
| Human-GAPDH | GTCTCCTCTGACTTCAACAGCG | ACCACCCTGTTGCTGTAGCCAA |
| Mouse-GAPDH | CATCACTGCCACCCAGAAGACTG | ATGCCAGTGAGCTTCCCGTTCAG |

**Table. S2 Antibodies used in this study**

| **Target**  **protein** | **Source** | **Catalog No.** | **Dilution**  **Concentration** | **Application** |
| --- | --- | --- | --- | --- |
| SLC7A5 | GeneTex | GTX638556 | 1:100/1:1000 | IHC/WB |
| CD4 | Cell Signaling | 48274 | 1:1000 | IHC |
| CD4 | Abcam | ab183685 | 1:2000 | mIHC |
| CD8 | Cell Signaling | 70306 | 1:200 | IHC |
| CD8 | Abcam | ab209775 | 1:2000 | mIHC |
| Foxp3 | Cell Signaling | 98377 | 1:100 | IHC |
| Foxp3 | Abcam | ab253297 | 1:1000 | mIHC |
| iNOS | Abcam | ab283655 | 1:1000 | IHC |
| CD163 | Selleck | F1548 | 1:500 | IHC |
| Ki-67 | Abcam | ab16667 | 1:200 | IHC |
| E-cadherin | Abmart | TA0131 | 1:100/1:1000 | IHC/WB |
| N-cadherin | Abmart | T55015 | 1:100/1:1000 | IHC/WB |
| Vimentin | Abclonal | A19607 | 1:1600/1:5000 | IHC/WB |
| TDO2 | Abcam | ab259359 | 1:1000 | WB |
| IDO1 | Proteintech | 13268-1-AP | 1:1000 | WB |
| AHR | Abclonal | A1451 | 1:200/1:2000 | IF/WB |
| CYP1B1 | Abcam | ab185954 | 1:20000 | WB |
| Lamin B1 | Proteintech | 12987-1-AP | 1:20000 | WB |
| IL-17 | Proteintech | 26163-1-AP | 1:200 | mIHC |
| HLA-DPB1 | Abclonal | A25794 | 1:2000 | mIHC |
| CD4 | Abcam | ab183685 | 1:2000 | mIHC |
| F4/80 | Cell Signaling | 70076S | 1:200 | mIHC |
| TIGIT | Abcam | ab300073 | 1:500 | IHC/mIHC |
| CTLA-4 | Abclonal | A2063 | 1:200 | IHC |
| VEGFA | Abcam | ab52917 | 1:100 | IHC |
| CD79a | Abcam | ab300150 | 1:5000 | IHC |
| GAPDH | Proteintech | 10494-1-AP | 1:4000 | WB |
| F4/80 | BioLegend | 111603 | / | FC |
| CD86 | BioLegend | 105005 | / | FC |
| CD163 | BioLegend | 155305 | / | FC |
| CD8 | BioLegend | 162313 | / | FC |
| PD-1 | BioLegend | 135205 | / | FC |
| LAG3 | BioLegend | 125211 | / | FC |

**Table. S3 shRNA and sgRNA sequences used in this study**

| **Target** | **Primer/Sequence (5′→3′)** |
| --- | --- |
| Human-shRNA-NC | TTCTCCGAACGTGTCACGT |
| Human-shRNA-SLC7A5-1 | CTAGATCCCAACTTCTCATTT |
| Human-shRNA-SLC7A5-2 | GCATTATACAGCGGCCTCTTT |
| Human-shRNA-SLC7A5-3 | GCCGTGGACTTCGGGAACTAT |
| Mouse-shRNA-NC | TTCTCCGAACGTGTCACGT |
| Mouse-shRNA-SLC7A5-1 | GGATCGAGCTGCTCATCATTC |
| Mouse-shRNA-SLC7A5-2 | GCTGACGAACCTGGCCTATTT |
| Mouse-shRNA-SLC7A5-3 | GTACGCCTTCTCCAGAGATAT |
| Human-sgRNA-Control | ACGGAGGCTAAGCGTCGCAA |
| Human-sgRNA-AhR-1 | GACCAGGGTCAAAATTGGGCT |
| Human-sgRNA-AhR-2 | GCAAAAACCAGGGTCAAAATT |
